# Supplementary material for: CXCR4 engagement triggers CD47 internalization and antitumor immunization in a mouse model of mesothelioma
Source: EMBO Mol Med. 2021 May 6;13(6):e12344. doi: 10.15252/emmm.202012344 (PMC8185548; doi:10.15252/emmm.202012344)

Appendix of the research article entitled

CXCR4 engagement triggers CD47 internalization and antitumor immunization in a mouse model of mesothelioma

Mezzapelle et al.

Table of content:

Appendix Fig. S1. Depletion $CD8^+$ T cells.

Appendix figure legend:

Appendix Figure S1. Depletion CD8+ T cells. Flow cytometry analysis shows the percentage of $CD8^+$ T cells in the spleen of a control mouse and a mouse treated with αCD8 antibody (Clone 2.43).

Appendix Figure S1

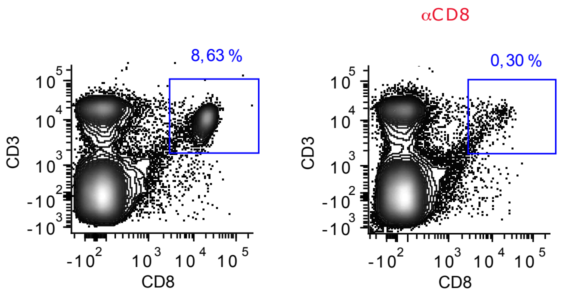

Supplement: Supplementary file 1 — Appendix [file EMMM-13-e12344-s006.pdf]
