# Supplementary material for: CXCR4 engagement triggers CD47 internalization and antitumor immunization in a mouse model of mesothelioma
Source: EMBO Mol Med. 2021 May 6;13(6):e12344. doi: 10.15252/emmm.202012344 (PMC8185548; doi:10.15252/emmm.202012344)
Supplement: Supplementary file 2 — Expanded View Figures PDF [file EMMM-13-e12344-s002.pdf]

Expanded View Figures

Figure EV1. Immunohistochemical survey of tumor microenvironment in mesothelioma.

A Representative IHC staining for F4/80, CD45R, CD3, and HMGB1 in two mouse mesotheliomas. Nuclei were counterstained with hematoxylin. Scale bar 20 µm.
B Representative immunofluorescence (IF) staining for F4/80 (red), CD86 (green, in the upper panel), and CD206 (green, in the lower panel) of mouse MM lesions. Scale bar 50 µm.
C Representative IHC staining for CD68, CD206, CD163, CD20, CD3, and HMGB1 in human sarcomatoid and epithelioid mesotheliomas. Scale bar 50 µm.

 © 2021 The Authors

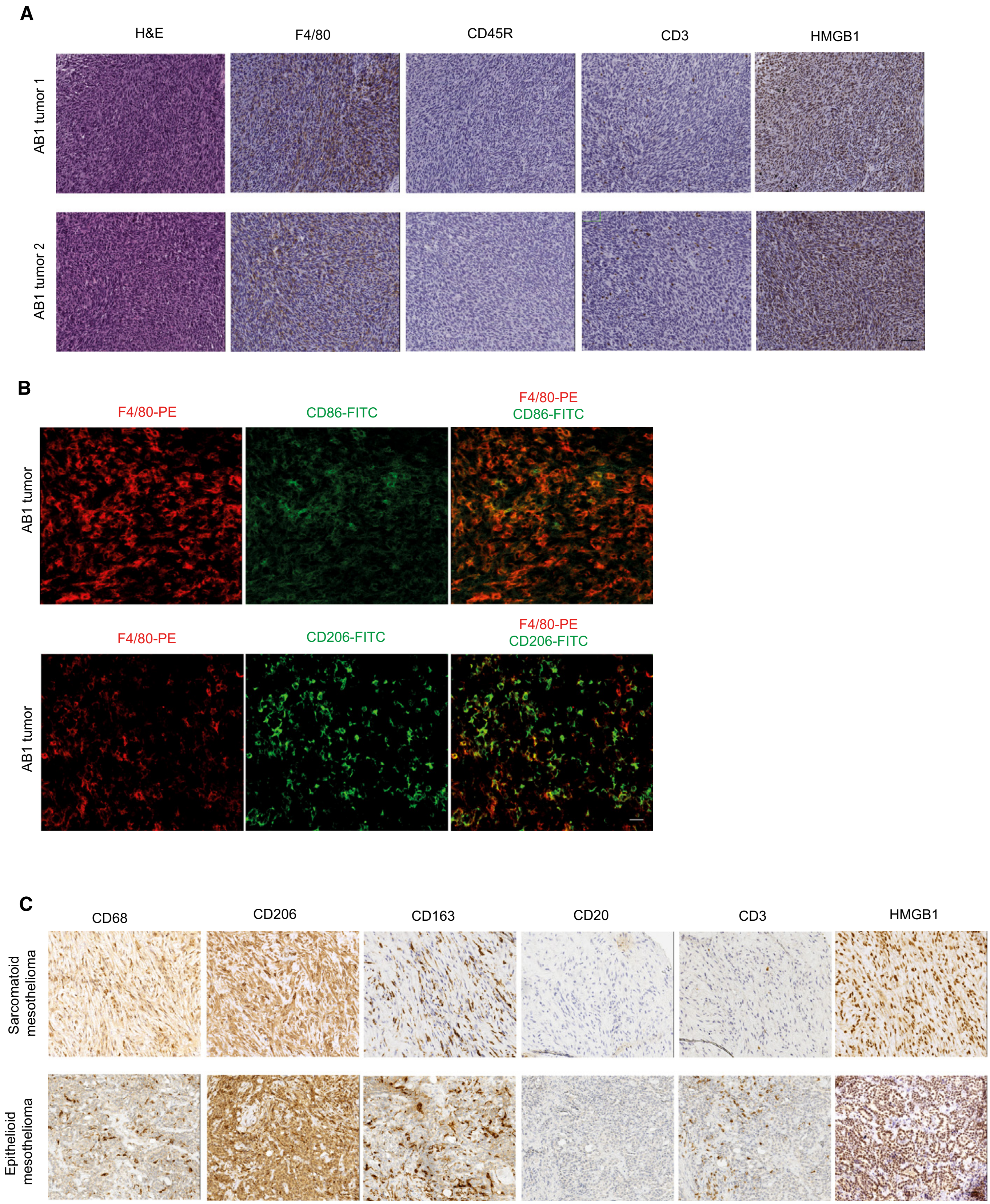
A
H&E
F4/80
CD45R
CD3
HMGB1
AB1 tumor 1
AB1 tumor 2
B
F4/80-PE
CD86-FITC
F4/80-PE
CD86-FITC
AB1 tumor
F4/80-PE
CD206-FITC
F4/80-PE
CD206-FITC
AB1 tumor
C
CD68
CD206
CD163
CD20
CD3
HMGB1
Sarcomatoid mesothelioma
Epithelioid mesothelioma

Figure EV1.

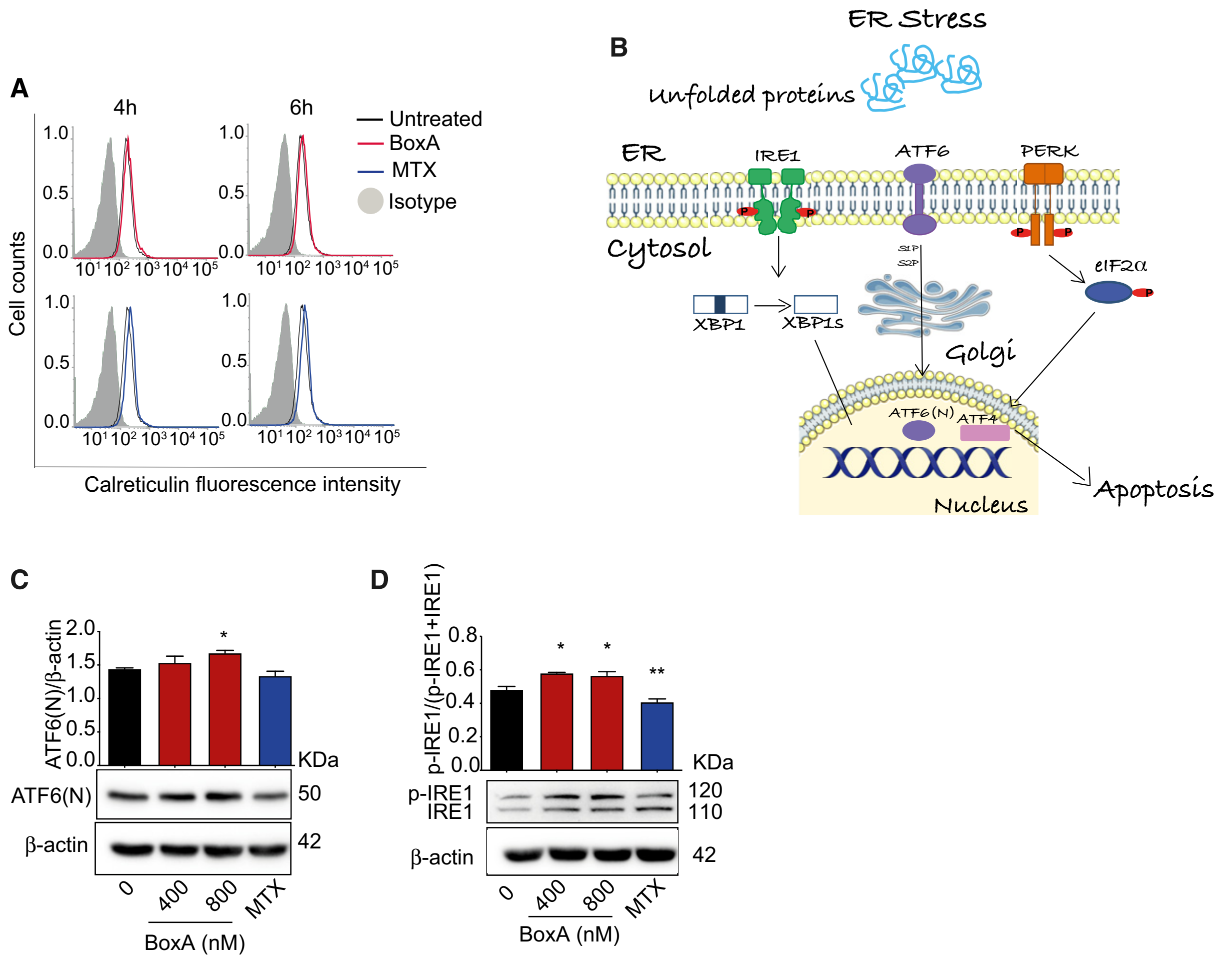

Figure EV2. BoxA induces the complete Unfolded Protein Response (UPR) in MM cells.

A Flow cytometry analysis of ecto-calreticulin on the surface of MM cells exposed to 800 nM BoxA (red line) or 1 μM MTX (blue line) for 4 or 6 h, compared to untreated cells (black line); control isotype is shown in gray.

B Schematic representation of the three branches of the UPR.

C Western blot analysis and quantification of the nuclear form of ATF6(*N*) relative to β-actin in MM cells treated with BoxA (400 and 800 nM) or MTX for 24 h.

D Western blot analysis of p-IRE1 relative to the total IRE1 in MM cells treated as in (C). β-actin is shown as loading control.

Data information: The experiments shown are representative of 2 performed. Error bars indicate standard deviation. Statistics (one-way ANOVA plus Dunnett's post-test) refers to pooled data from two experiments. $^*P < 0.05$, $^{**}P < 0.01$.
Source data are available online for this figure.

 © 2021 The Authors

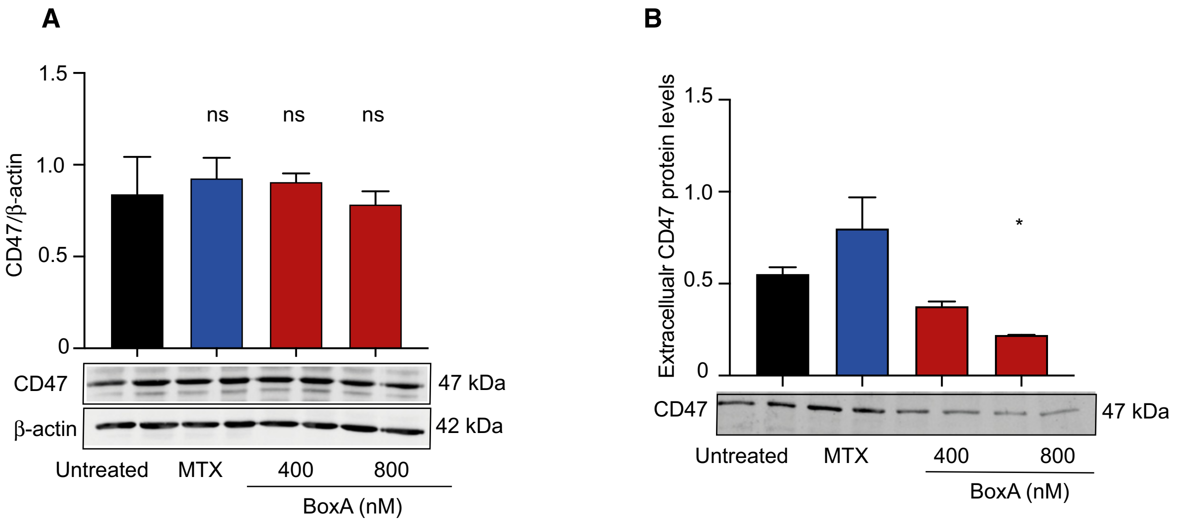

Figure EV3. BoxA does not induce CD47 degradation or shedding.

A Western blot analysis of CD47 in the total lysate of MM cells exposed to BoxA or 1 μM MTX for 24 h. β-actin was used for normalization.

B Western blot of CD47 assessed in the medium of MM cells exposed to BoxA for 24 h. Ponceau staining was used as loading control.

Data information: The experiments shown are representative of 2 performed, in biological duplicate. Error bars indicate standard deviation. Statistics: one-way ANOVA plus Dunnett's post-test, $*P < 0.05$.
Source data are available online for this figure.

Figure EV4. BoxA induces DAMPs release and reduces surface CD47 in different tumor cell lines.

A Surface CD47 on MC38, B16, and U87 cells evaluated by flow cytometry after incubation for 24 or 48 h with 800 nM BoxA. The experiments shown are representative of 2 performed, in biological triplicate or quadruplicate. Bars and error bars represent mean ± SD. Statistics: *t*-test.

B CD47 surface exposure. Independent cultures of LoVo cells ($n = 4$) were exposed or not to 800 nM BoxA for 48 h. The distribution of CD47 fluorescence intensity is normalized by indicating as 1.0 the fluorescence of the most abundant cells in the population: control isotype (gray), cells treated (red) or not (black) with BoxA. Bars and error bars represent mean ± SD. The MFI of the different samples is compared by paired *t*-test.

C Translocation of calreticulin: representative flow cytometry analysis of ecto-calreticulin. LoVo cells untreated (black) or treated with 800 nM BoxA (red) for 8 and 12 h. Control isotype (gray).

D Western blot analysis of extracellular HMGB1 in the medium of LoVo cells exposed for 24 and 48 h to 800 nM BoxA ($n = 2$). Ponceau S staining was used for protein loading normalization. Bars and error bars represent mean ± SD. Statistics: one-way ANOVA plus Dunnett's post-test.

E Western blot analysis of p-eIF2α, the nuclear form of ATF6, ATF6(N), and p-IRE-1 in LoVo cells treated with 400 or 800 nM BoxA for 48 h. β-actin was used for normalization in the case of ATF6(N), while only as protein loading control for p-IRE1 and p-eIF2α. Bars and error bars represent mean ± SD ($n = 2$). Statistics: one-way ANOVA plus Dunnett's post-test.

Data information: $*P < 0.05$, $**P < 0.01$, $***P < 0.001$, $****P < 0.0001$.
Source data are available online for this figure.

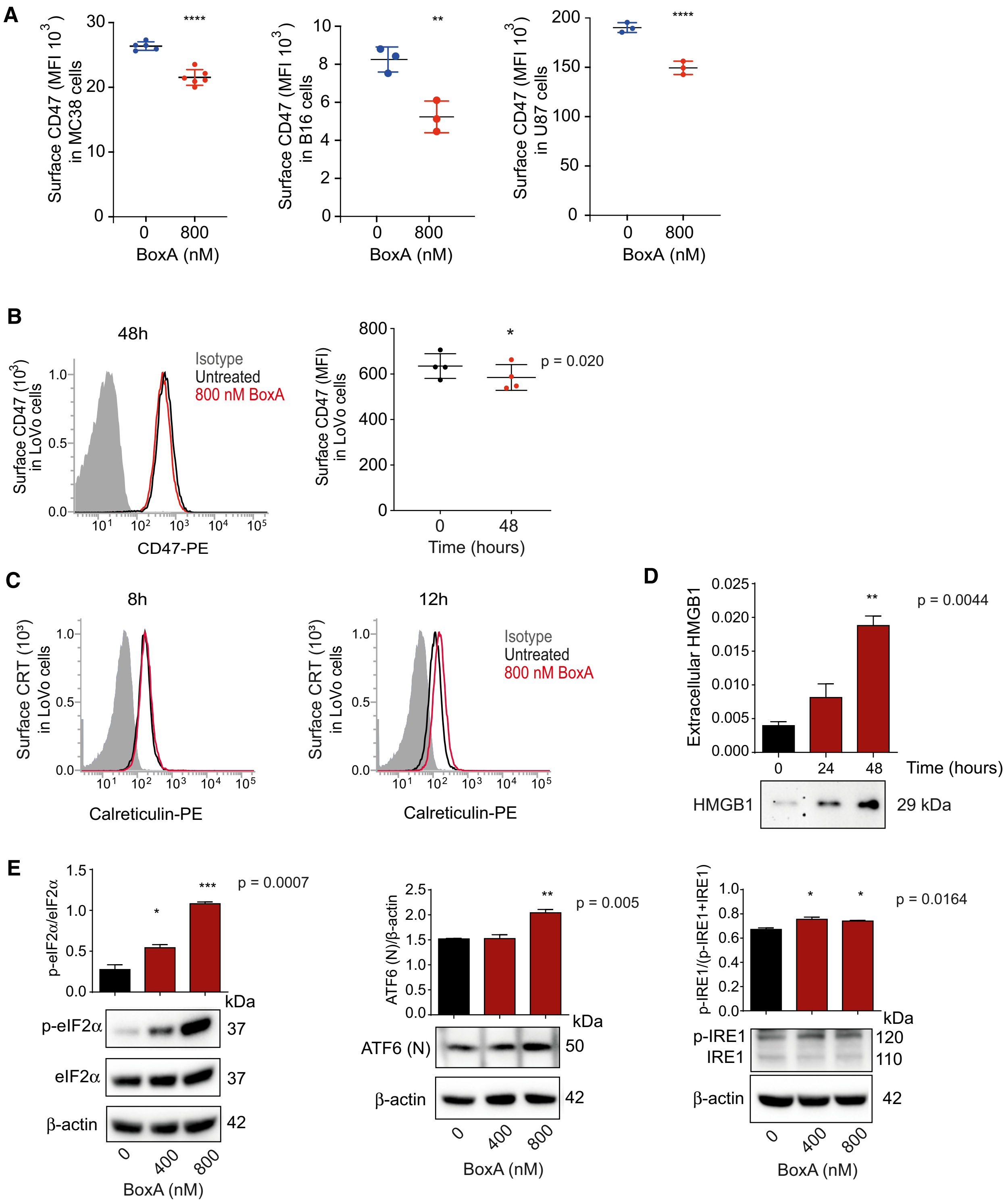

Figure EV4.

 © 2021 The Authors

Figure EV5. BoxA exerts therapeutic effects in a syngeneic model of colon cancer.

A Flow cytometry analysis of ecto-calreticulin on the surface of CT26 cells exposed to 800 nM BoxA (red line) or 1 μM MTX (blue line) for 2 or 4 h, compared to untreated cells (black line); control isotype is shown in gray.

B Western blot and quantification of the nuclear form of ATF6(N) relative to β-actin in CT26 cells treated with BoxA (400 and 800 nM) or MTX (1 μM) for 24 h. The experiments shown are representative of three performed. Error bars indicate standard deviation. Statistics (one-way ANOVA plus Dunnett's post-test) refers to pooled data from the three experiments. $*P < 0.05$.

C Western blot of p-IRE1 relative to total IRE1 in CT26 cells treated as in B. β-actin is shown as loading control. The experiments shown are representative of three performed. Error bars indicate standard deviation. Statistics (one-way ANOVA plus Dunnett's post-test) refers to pooled data from the three experiments. $*P < 0.05$, $**P < 0.01$.

D Confocal immunofluorescence microscopy of cleaved caspase-3 (red) in CT26 cells treated with increasing doses of BoxA or MTX (1 μM) for 24 h. Nuclei were stained with Hoechst (blue) and cytosol with phalloidin (green). Scale bar 50 μm.

E Tumor growth followed by BLI in mice (n = 3 per group) injected i.p. with three different amounts of CT26-LUC cells.

F Necroscopy of a mouse inoculated with 3.5×10^4 CT26-LUC cells (19 days post-injection). White arrows and circles indicate tumor masses.

G Immunohistochemical survey of tumor microenvironment in CT26 colon cancers explanted from 2 mice; representative H&E and IHC staining. Scale bar, 200 μm.

Source data are available online for this figure.

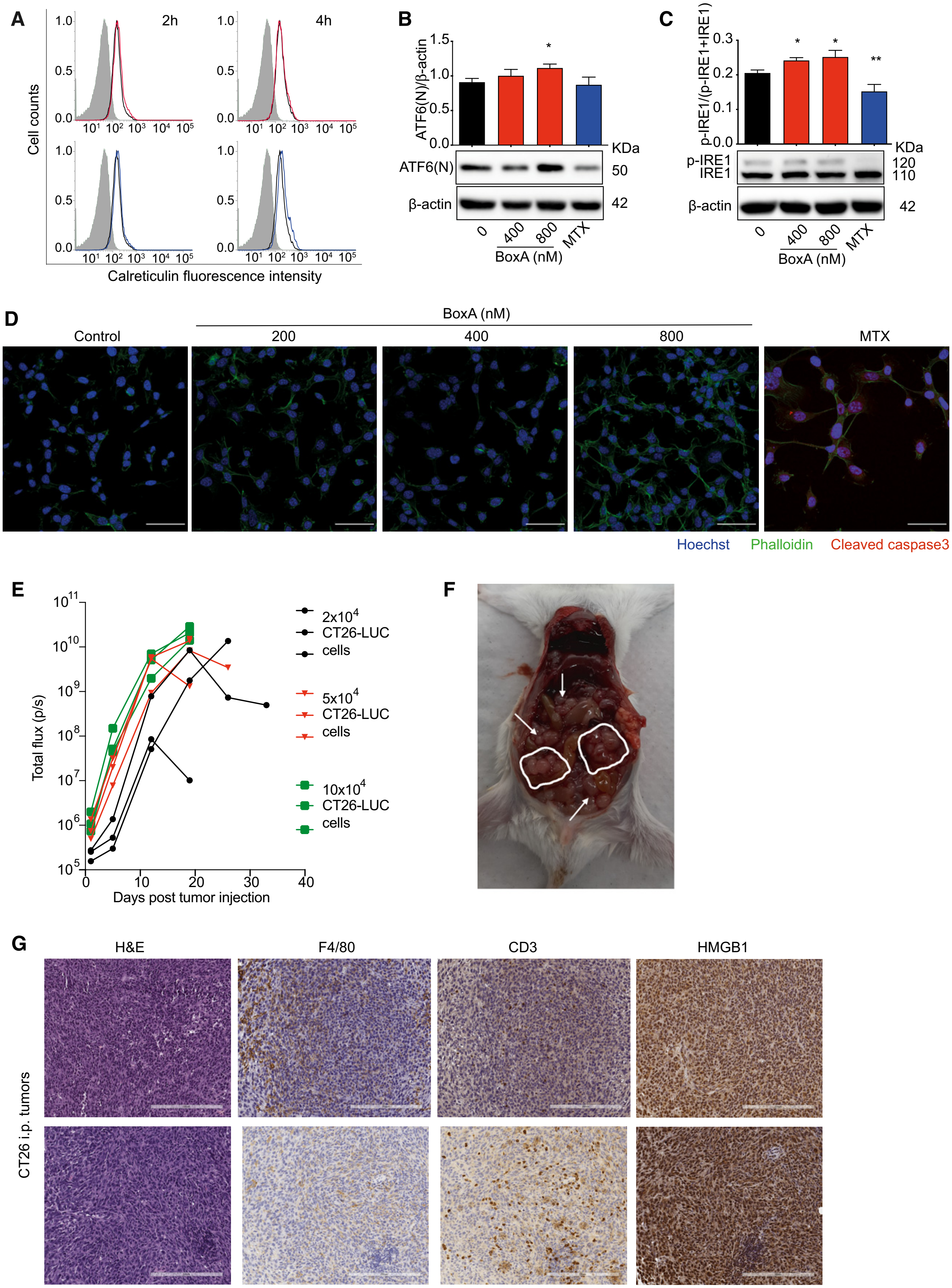

Figure EV5.

 © 2021 The Authors

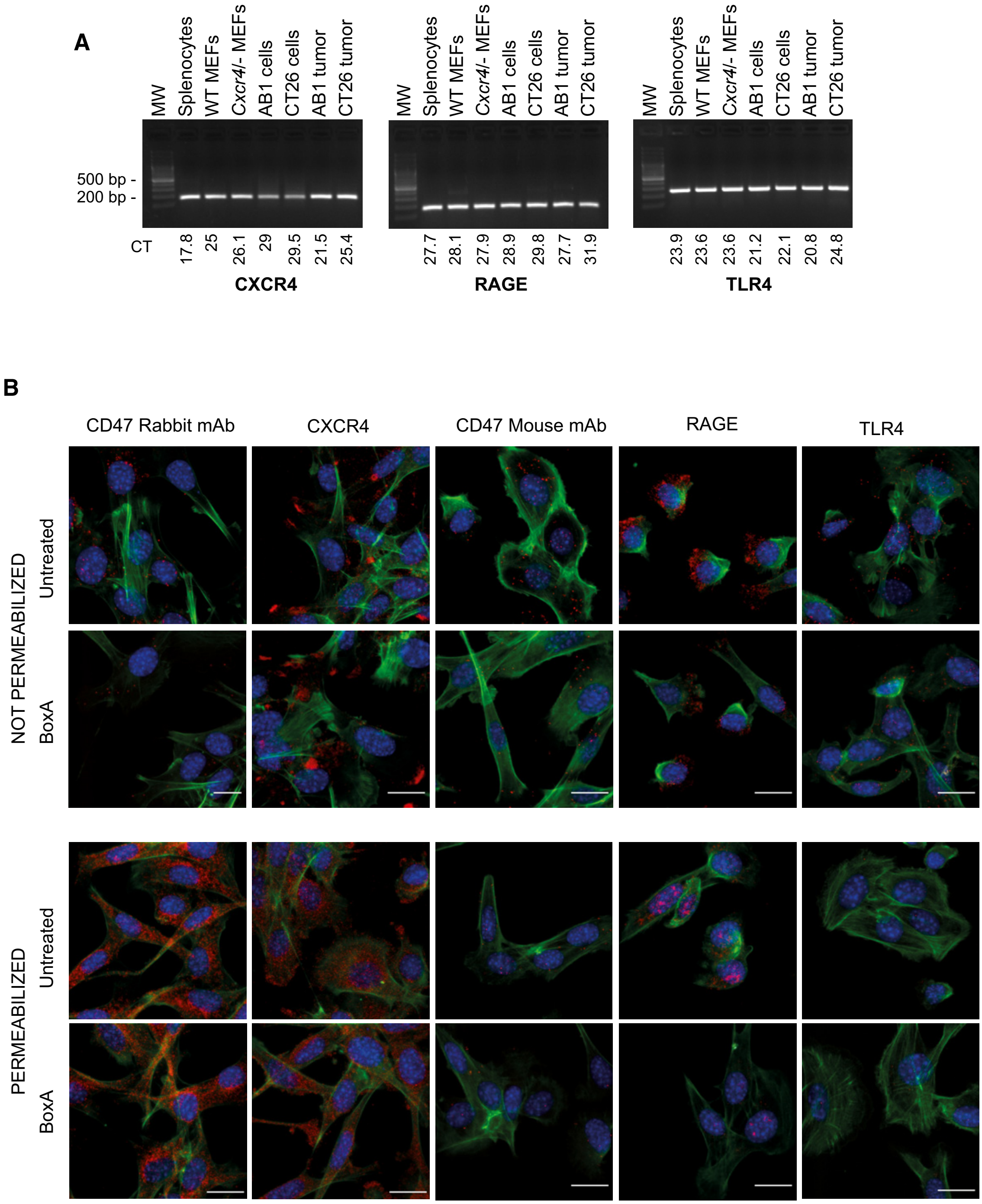

Figure EV6. Expression of CXCR4, RAGE, TLR4, and CD47 molecules.

A mRNAs extracted from the indicated samples were subjected to qPCR with primers designed on *Cxcr4*, *Rage*, and *Tlr4* transcripts. PCR products were loaded on a 2% agarose gel. Numbers below the lanes indicated CT values for the various samples.

B Representative confocal image of Proximity Ligation Assay performed on CD47, CXCR4, RAGE, and TLR4. MM cells were incubated over night with 400 nM BoxA or PBS and then permeabilized or not. Red dots represent physical contact of two identical receptor molecules. Nuclei are in blue (DAPI), phalloidin is green. Scale bar, 20 μm. Representative images from one experiment out of three performed are shown.

Source data are available online for this figure.

© 2021 The Authors
