# Supplementary material for: CXCR4 engagement triggers CD47 internalization and antitumor immunization in a mouse model of mesothelioma
Source: EMBO Mol Med. 2021 May 6;13(6):e12344. doi: 10.15252/emmm.202012344 (PMC8185548; doi:10.15252/emmm.202012344)
Supplement: Supplementary file 3 — Movie EV1 [file EMMM-13-e12344-s009.zip › Legend to movie EV1.docx]

**Movie EV1**. **Tumor cell phagocytosis by macrophages.** Representative video of a 16 hour time-lapse microscopy experiment; GFP+ MM cells (green) were co-cultured with mouse bone marrow derived macrophages (grey) in the presence of 200 nM BoxA.
