# Supplementary material for: CXCR4 engagement triggers CD47 internalization and antitumor immunization in a mouse model of mesothelioma
Source: EMBO Mol Med. 2021 May 6;13(6):e12344. doi: 10.15252/emmm.202012344 (PMC8185548; doi:10.15252/emmm.202012344)
Supplement: Supplementary file 6 — Source Data for Figure 1 [file EMMM-13-e12344-s003.zip › Source of data Fig 1.pdf]

Figure 1 E
BLI original images from BLI scans

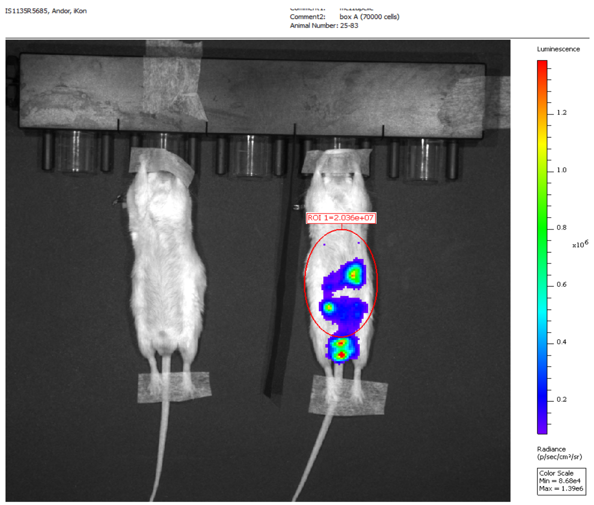

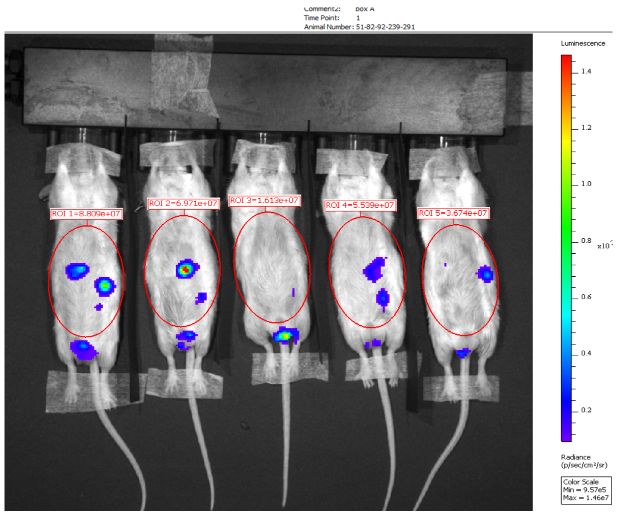

First scan at the re-challenge day 62.
First mouse on the left was not injected with AB1 LUC cells and was not included in the figure

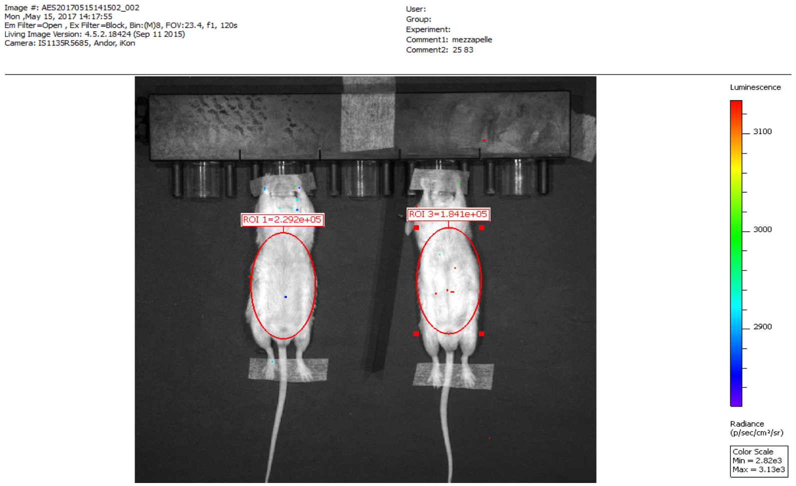

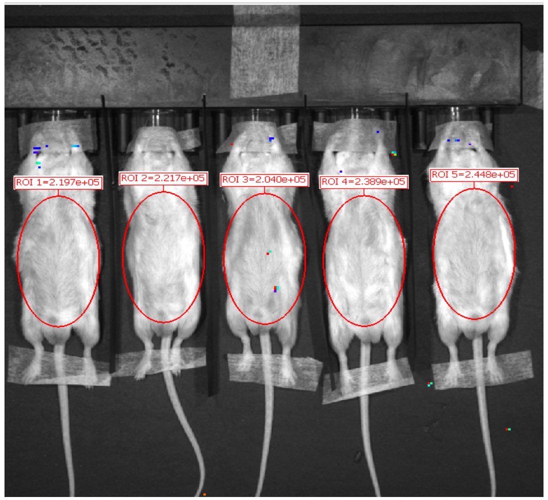

Second scan at the re-challenge day 69.
First mouse on the left was not injected with AB1 LUC cells and was not included in the figure

Figure 1 F

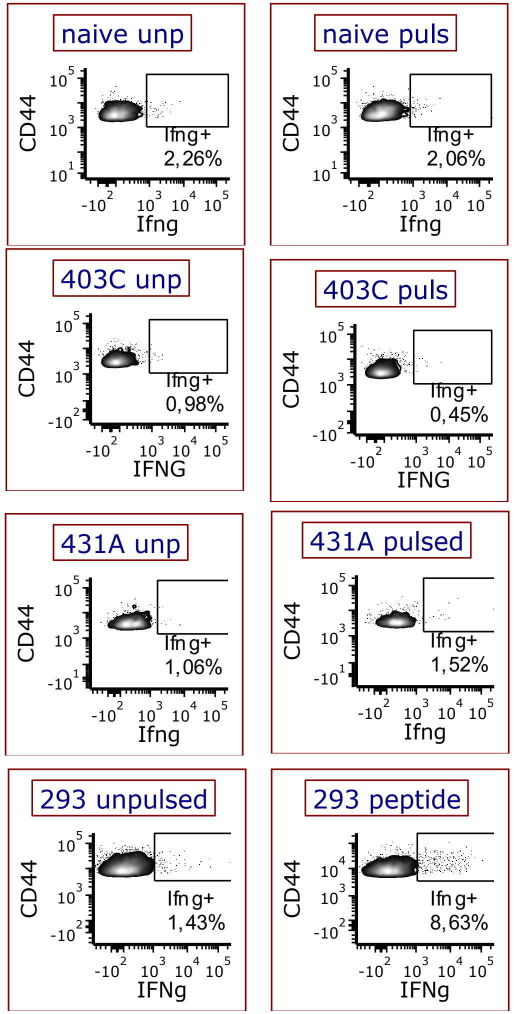

Figure 1 G

		% IFNγ+ in CD8+CD44high		
Treatment	Mouse #	unpulsed	Pulsed (peptide)	pulsed-unpulsed
naive	1	11,64	13,24	1,6
naive	2	11,98	6,92	-5,06
naive	3	5,24	7,53	2,29
naive	4	2,26	2,06	-0,2
naive	5	1,15	1,16	0,01
naive	6	0,68	0,46	-0,22
naive	7	0,25	0,11	-0,14
PBS	101	1,19	0,56	-0,63
PBS	274	2,89	1,08	-1,81
PBS	403	0,98	0,45	-0,53
PBS	45	1,64	1,51	-0,13
PBS	271	1	1,18	0,18
BOXA not cured	105	0,96	0,65	-0,31
BOXA not cured	270	1,25	0,92	-0,33
BOXA not cured	431	1,06	1,52	0,46
BOXA cured	35	0,63	5,1	4,47
BoxA cured	293	1,43	8,63	7,2
BOXA cured	NO earring	1,09	3,23	2,14
BOXA cured	291	2,07	27,86	25,79
BOXA cured	83	1,74	4,52	2,78

Unp and unpulsed stand for samples NOT stimulated with Luc peptide
Puls, pulsed and peptide stand for samples stimulated with Luc peptide
