# Supplementary material for: CXCR4 engagement triggers CD47 internalization and antitumor immunization in a mouse model of mesothelioma
Source: EMBO Mol Med. 2021 May 6;13(6):e12344. doi: 10.15252/emmm.202012344 (PMC8185548; doi:10.15252/emmm.202012344)
Supplement: Supplementary file 8 — Source Data for Figure 3 [file EMMM-13-e12344-s007.zip › Source of data Fig 3.pdf]

Figure 3

D

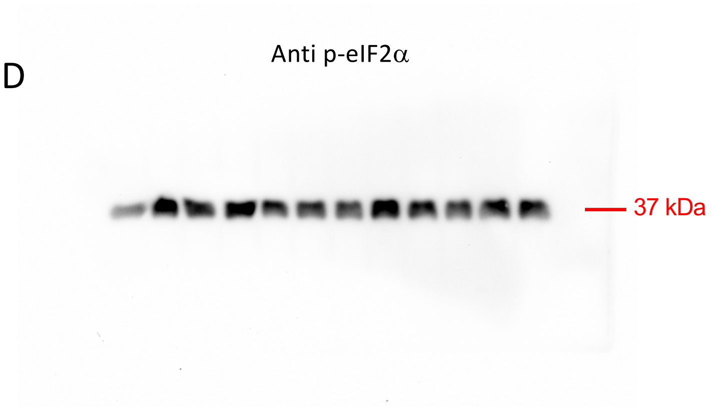

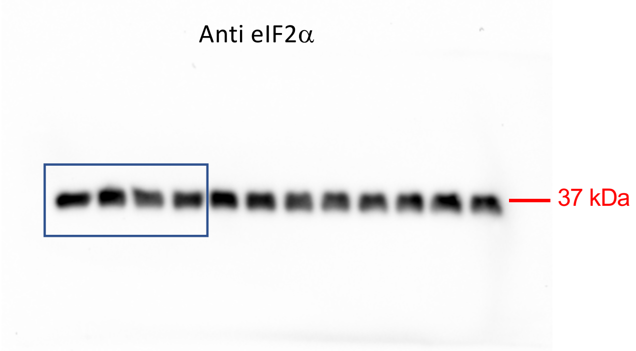

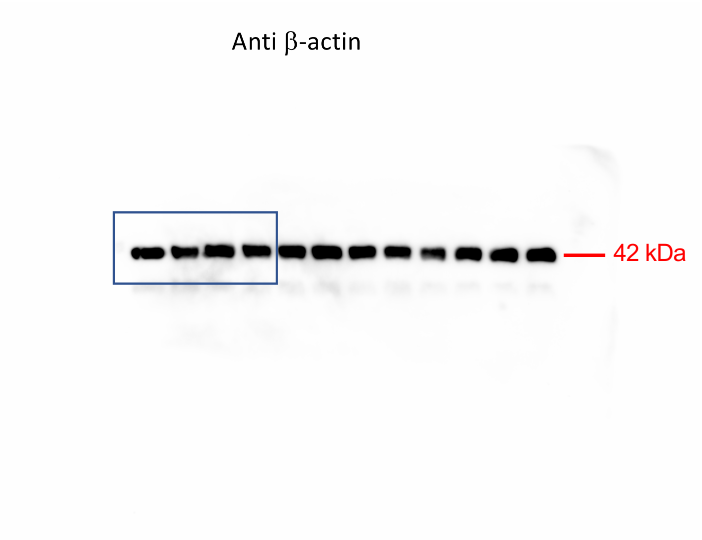

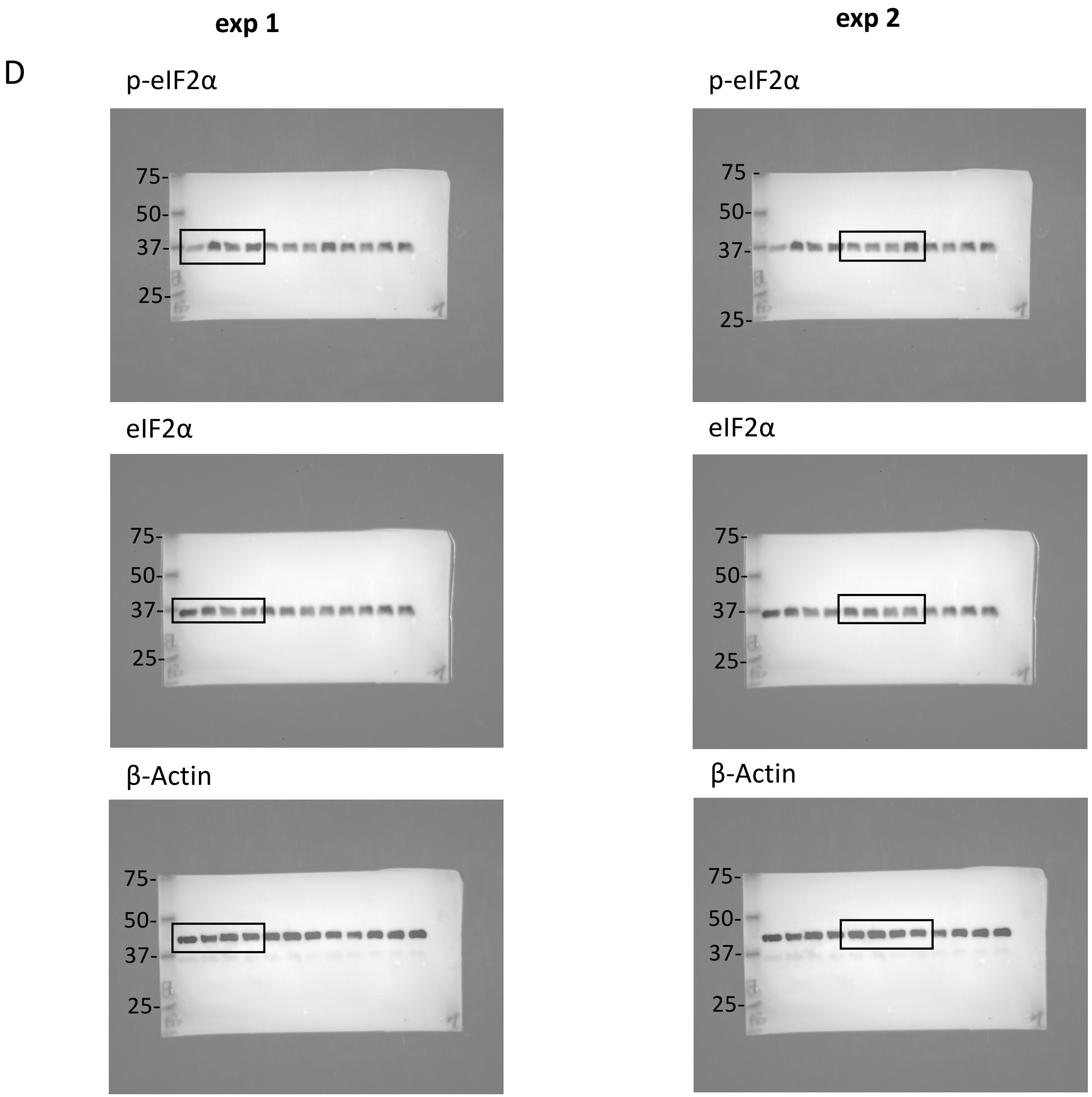

Exp 2 is not shown in the manuscript
but it was used for the statistical analysis

F

Anti cleaved caspase-3

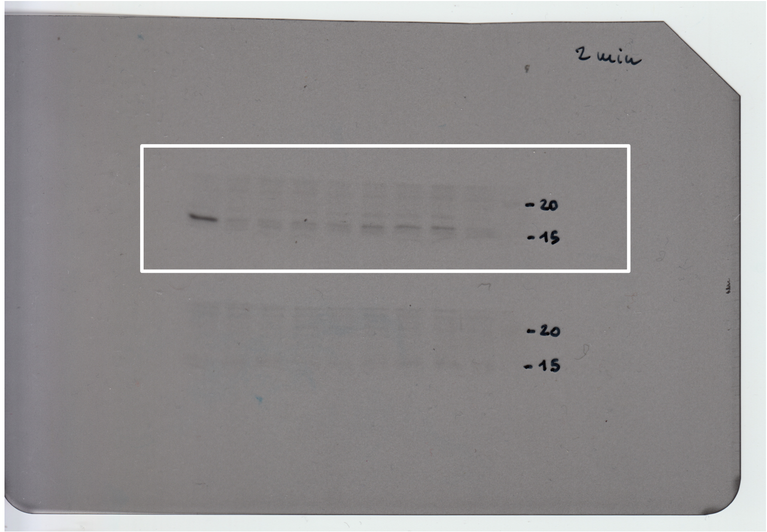

Anti α-tubulin

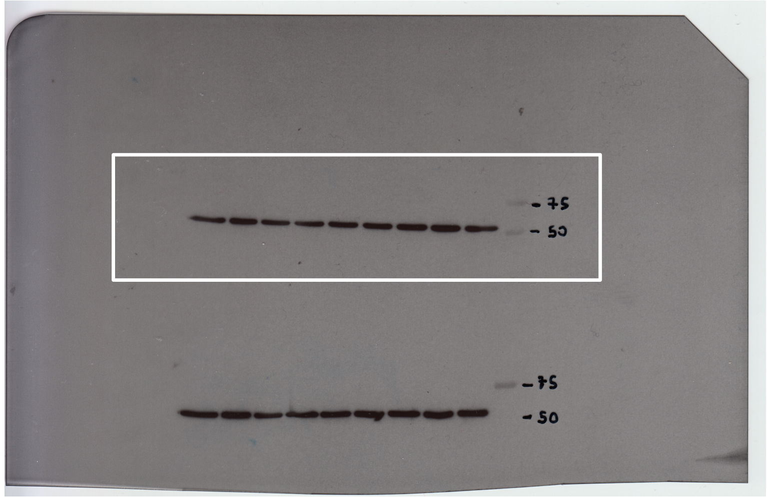

Images in the white rectangle were flipped orizontally in the figure
