# Supplementary material for: CXCR4 engagement triggers CD47 internalization and antitumor immunization in a mouse model of mesothelioma
Source: EMBO Mol Med. 2021 May 6;13(6):e12344. doi: 10.15252/emmm.202012344 (PMC8185548; doi:10.15252/emmm.202012344)
Supplement: Supplementary file 10 — Source Data for Figure 5 [file EMMM-13-e12344-s005.zip › Source of data Fig 5.pdf]

Figure 5

C

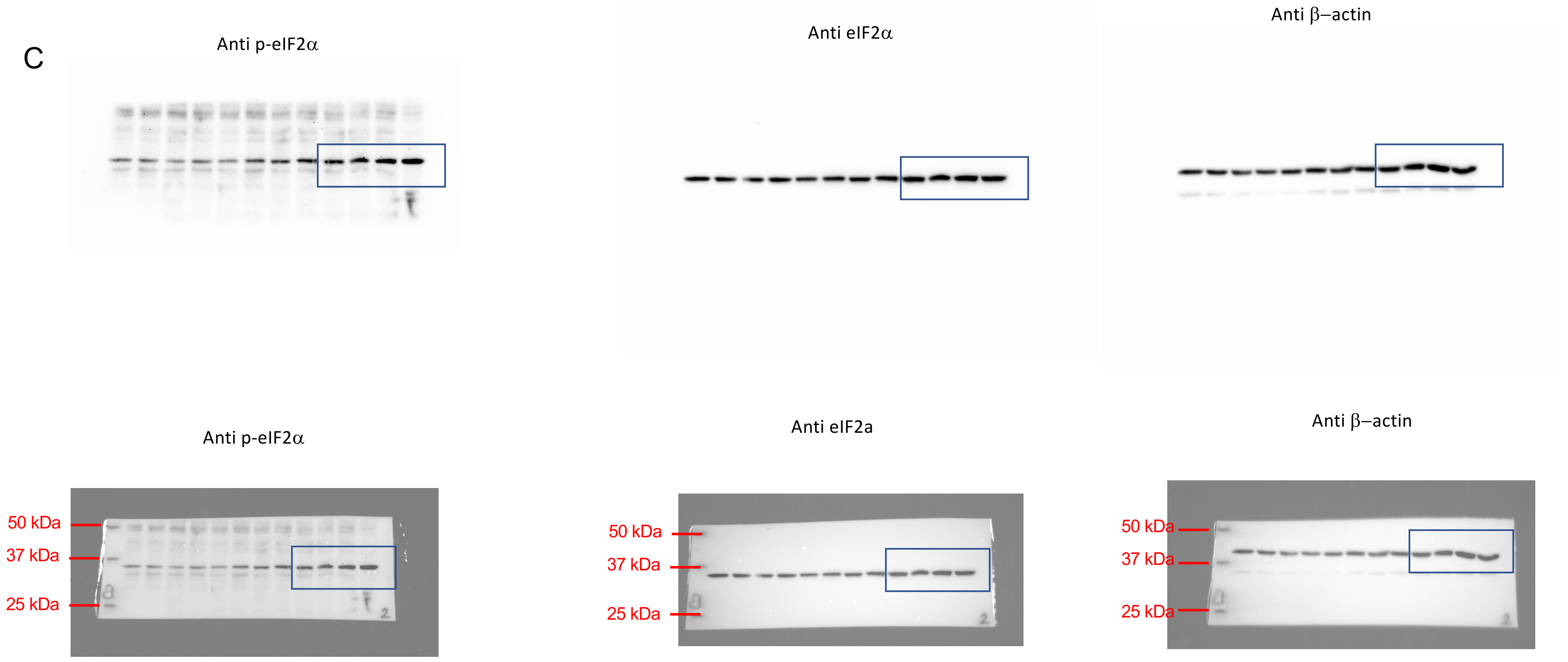

Anti p-eIF2α
Anti eIF2α
Anti β–actin
Anti p-eIF2α
50 kDa
37 kDa
25 kDa
Anti eIF2a
50 kDa
37 kDa
25 kDa
Anti β–actin
50 kDa
37 kDa
25 kDa

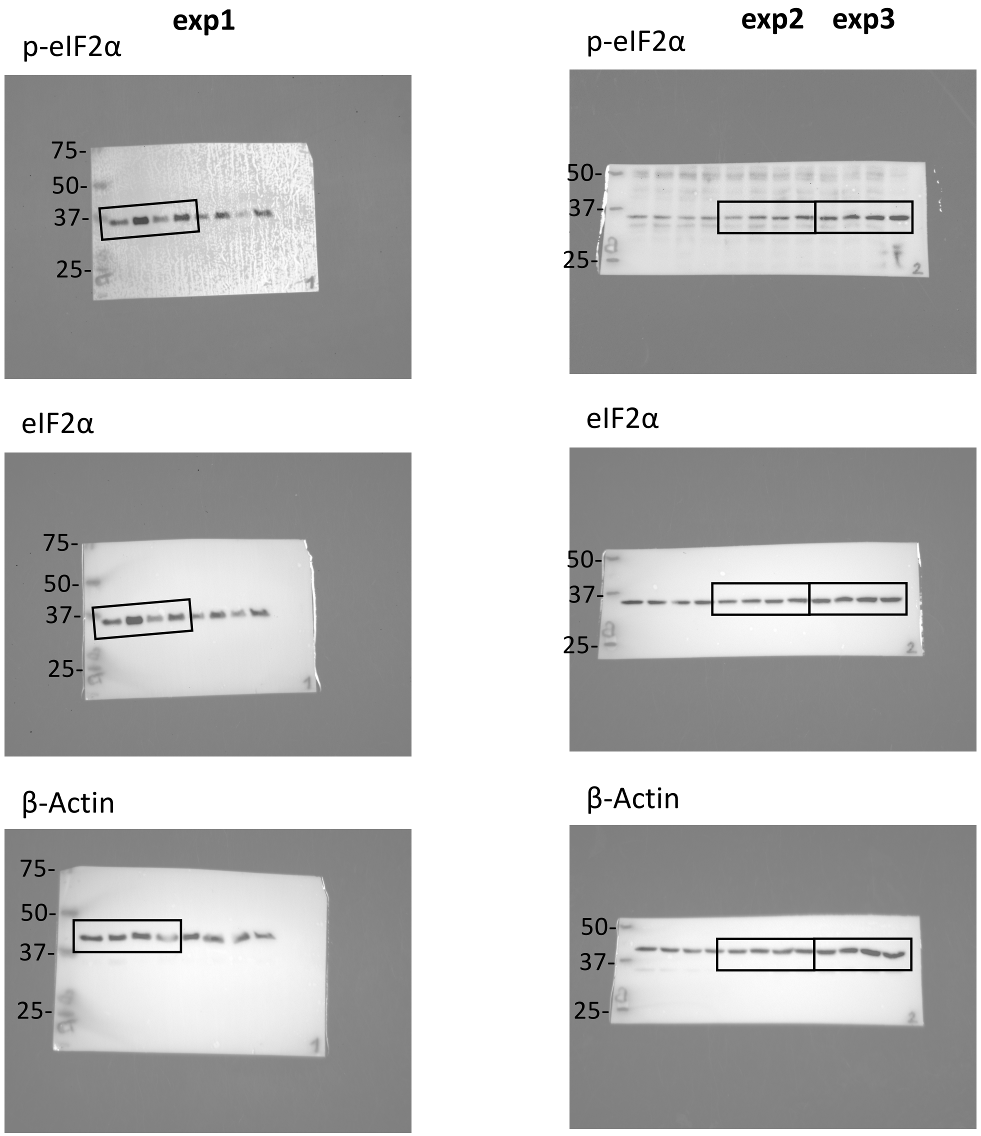

Exp1 and exp2 are not shown in the manuscript but they were used for the statistical analysis

Figure 5

F

Anti cleaved caspase-3

Anti α-tubulin

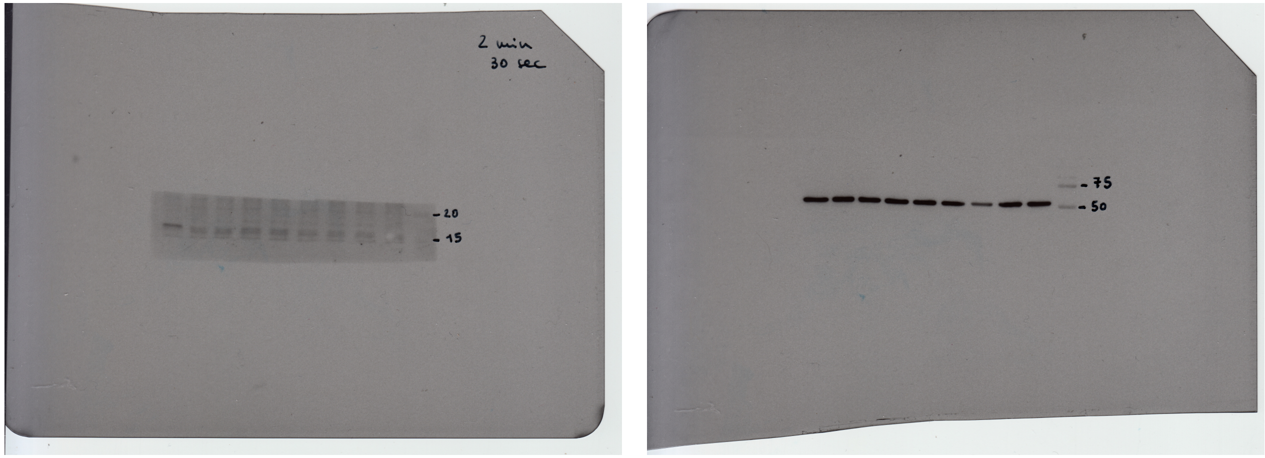

In the manuscript they were cropped and flipt orizontally

I

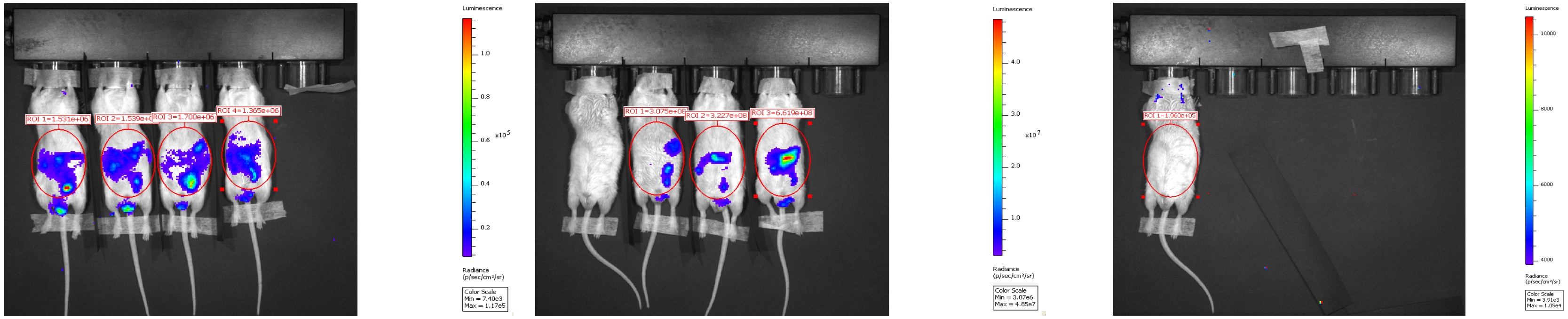

Scan day 65

Scan day 72
