# Supplementary material for: CXCR4 engagement triggers CD47 internalization and antitumor immunization in a mouse model of mesothelioma
Source: EMBO Mol Med. 2021 May 6;13(6):e12344. doi: 10.15252/emmm.202012344 (PMC8185548; doi:10.15252/emmm.202012344)

Figure 7

A

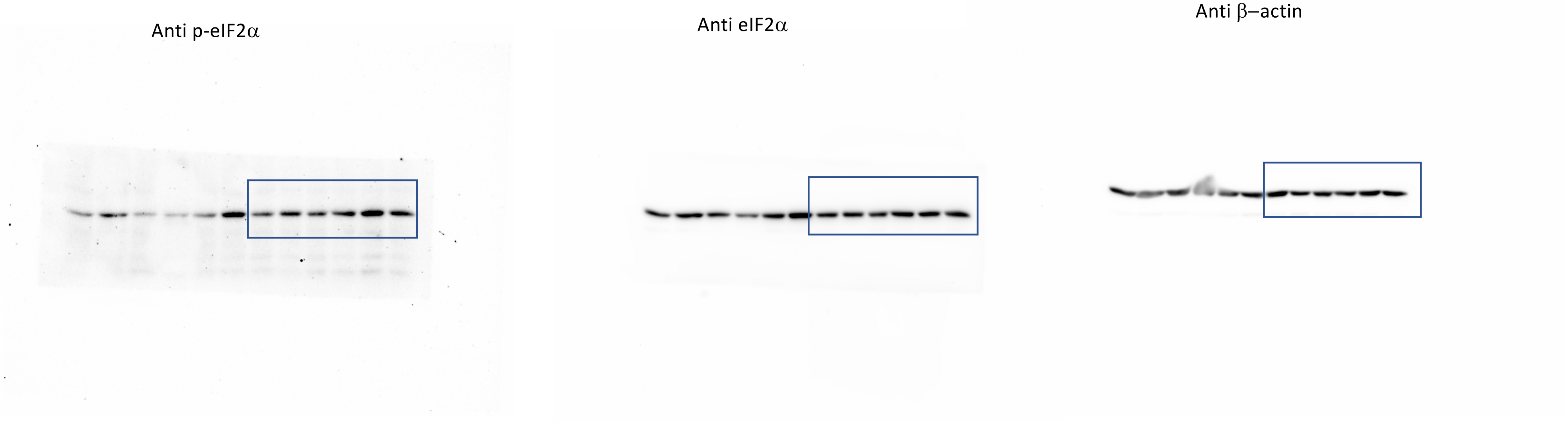

exp1

p-eIF2α

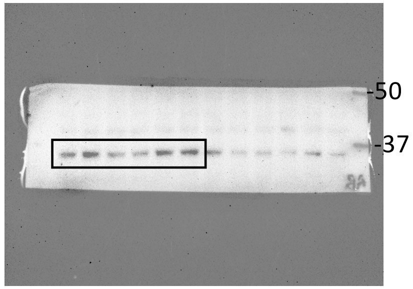

eIF2α

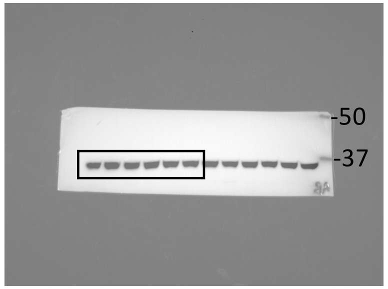

β-Actin

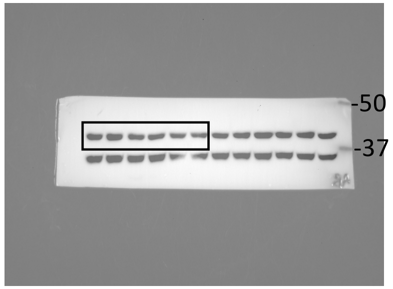

exp2

p-eIF2α

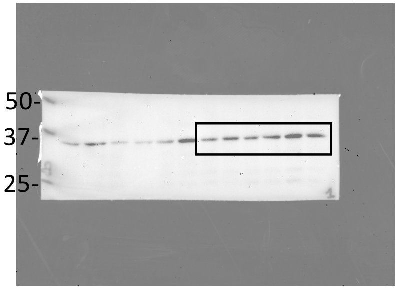

eIF2α

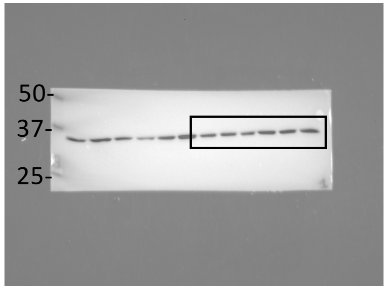

β-Actin

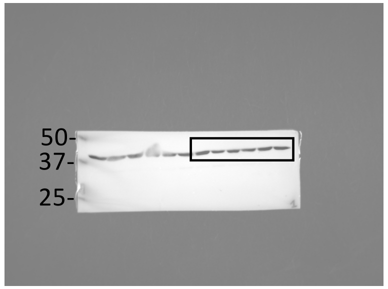

Exp1 is not shown in the manuscript but it was used for statistical analysis

Figure 7 Exp1 is not shown in the manuscript but it was used for statistical analysis

B

Exp 1

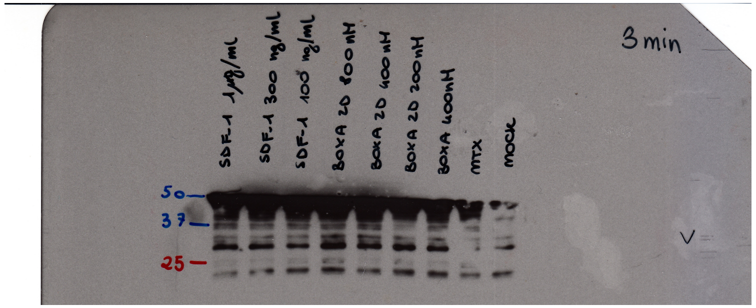

Exp 2

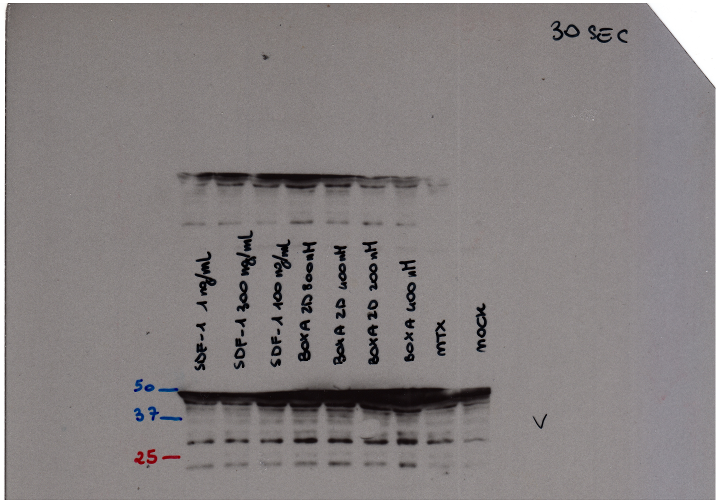

Ponceau staining

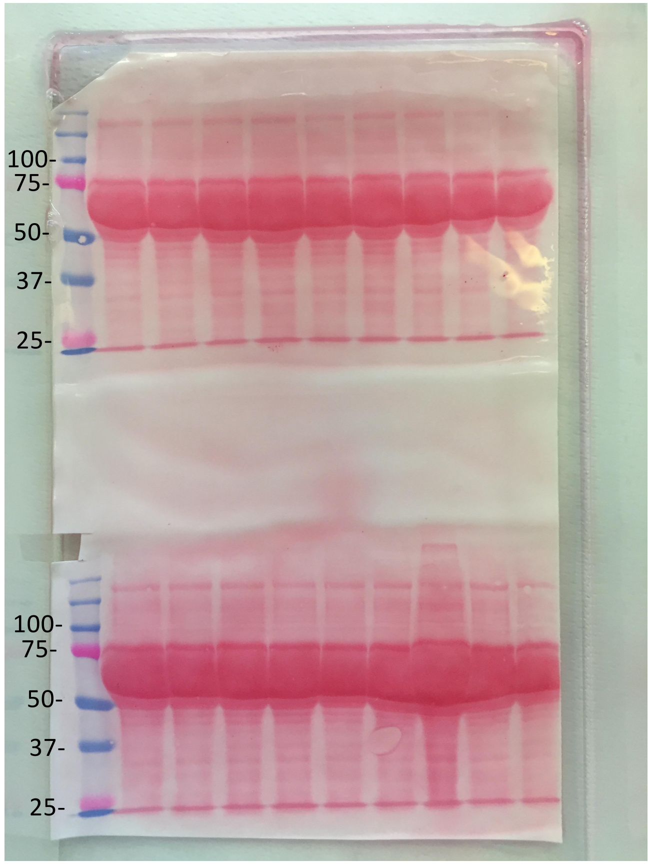

Supplement: Supplementary file 12 — Source Data for Figure 7 [file EMMM-13-e12344-s011.zip › Source of data Fig 7.pdf]
