# Supplementary material for: CXCR4 engagement triggers CD47 internalization and antitumor immunization in a mouse model of mesothelioma
Source: EMBO Mol Med. 2021 May 6;13(6):e12344. doi: 10.15252/emmm.202012344 (PMC8185548; doi:10.15252/emmm.202012344)

Figure EV2

C

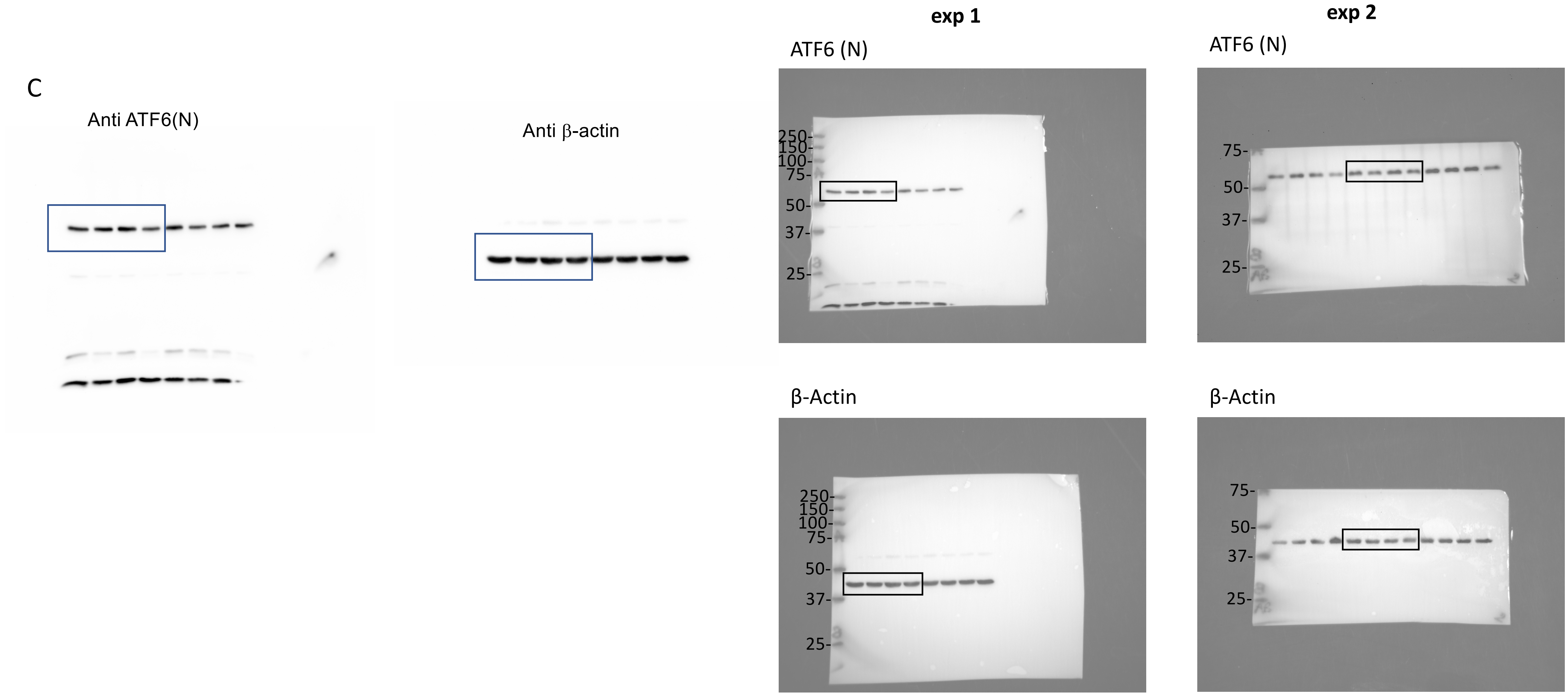

Exp 2 is not shown in the manuscript
but it was used for the statistical analysis

Figure EV2

D

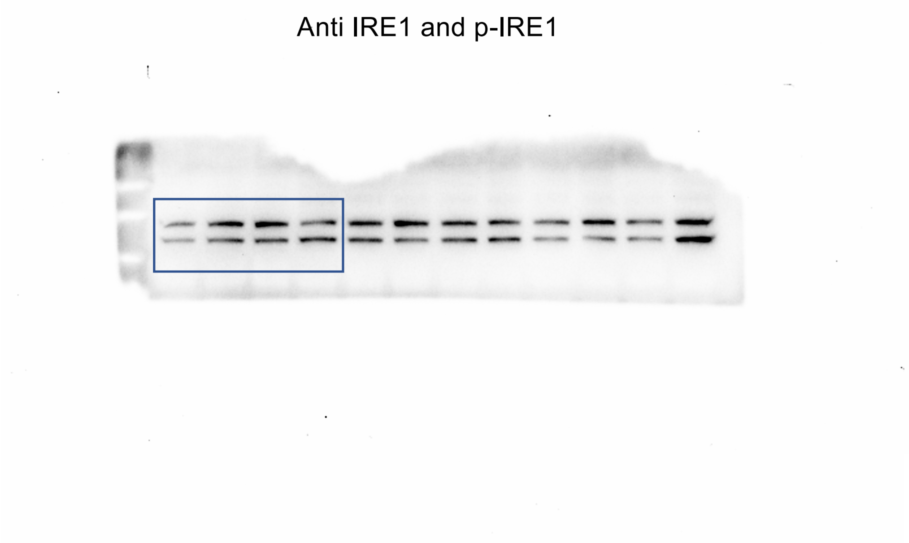

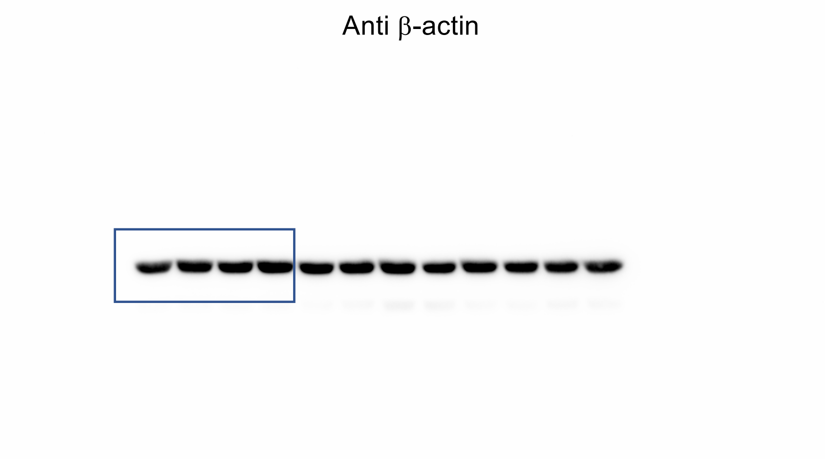

Exp1

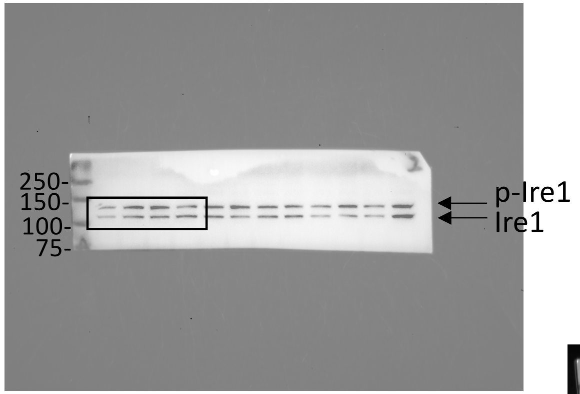

β-Actin

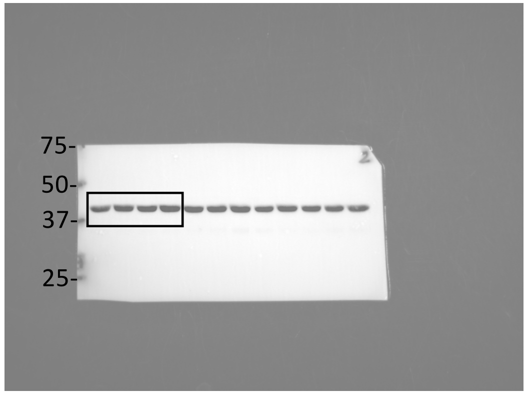

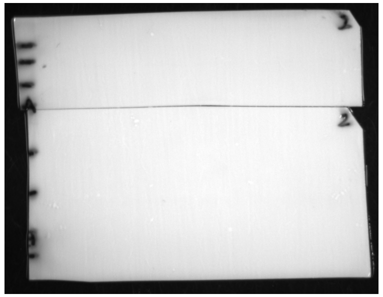

Exp2

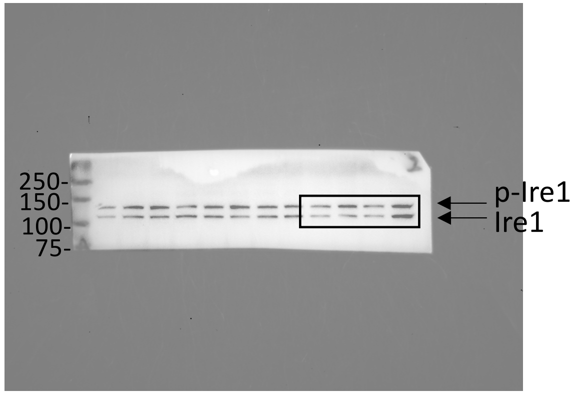

Exp 2 is not shown in the manuscript
but it was used for the statistical analysis

β-Actin

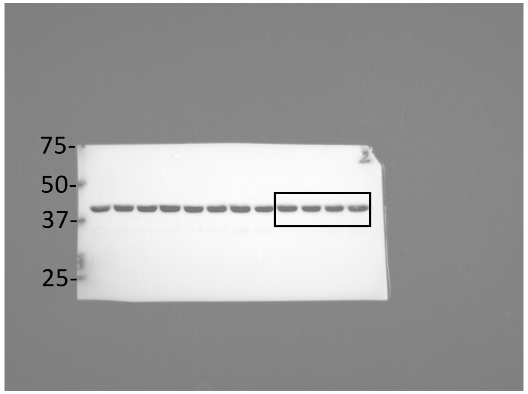

Supplement: Supplementary file 4 — Source Data for Expanded View and Appendix [file EMMM-13-e12344-s008.zip › EMM-2020-12344-V3-EV_Figures_SourceData/Source_of_data_EV2.zip!/Source of data EV2.pdf]
