# Supplementary material for: CXCR4 engagement triggers CD47 internalization and antitumor immunization in a mouse model of mesothelioma
Source: EMBO Mol Med. 2021 May 6;13(6):e12344. doi: 10.15252/emmm.202012344 (PMC8185548; doi:10.15252/emmm.202012344)
Supplement: Supplementary file 4 — Source Data for Expanded View and Appendix [file EMMM-13-e12344-s008.zip › EMM-2020-12344-V3-EV_Figures_SourceData/Source_of_data_EV3.zip!/Source of data EV3.pdf]

Figure EV3

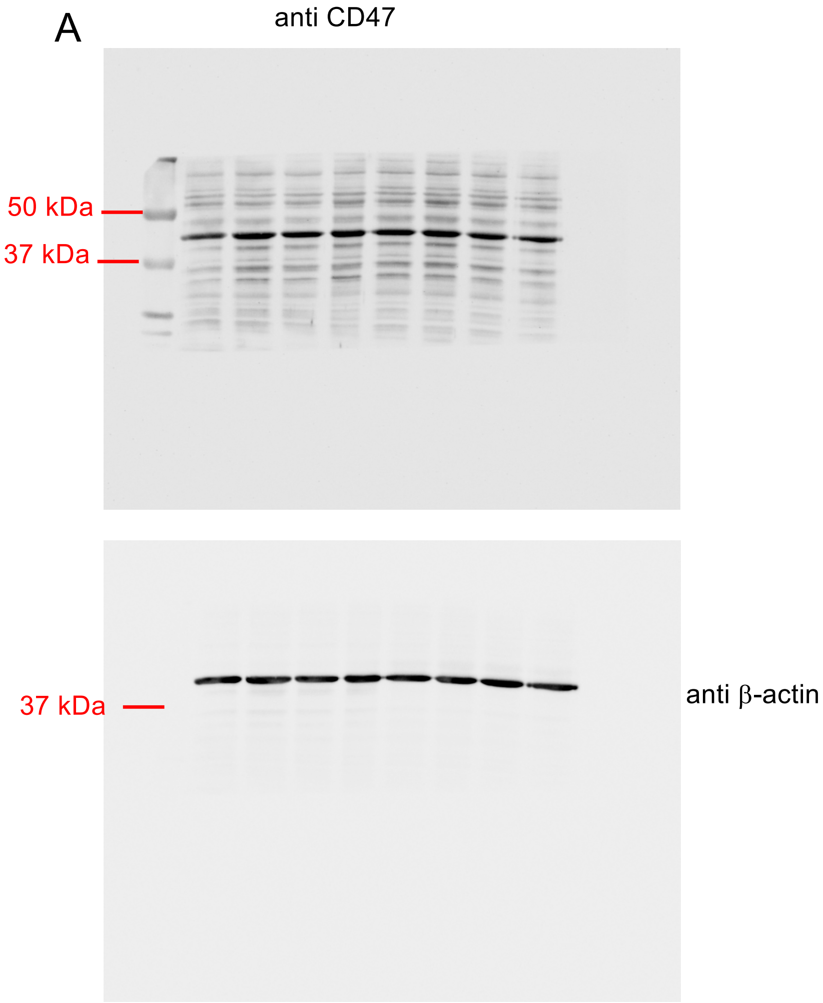
A
anti CD47
50 kDa
37 kDa
37 kDa
anti β-actin

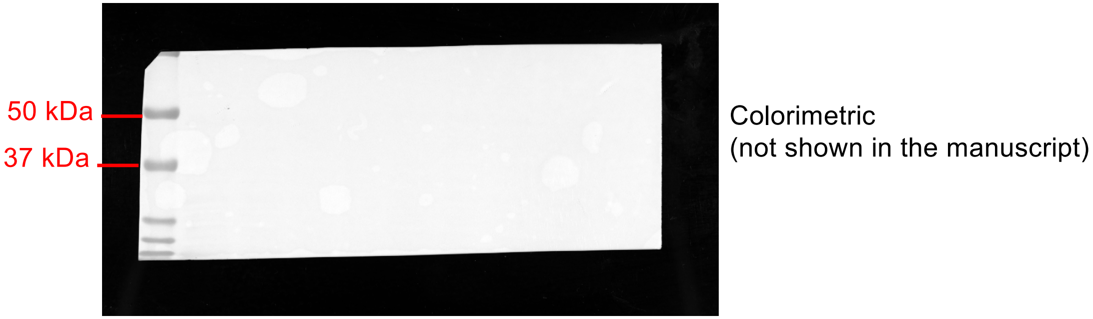
50 kDa
37 kDa
Colorimetric
(not shown in the manuscript)

Figure EV3

B

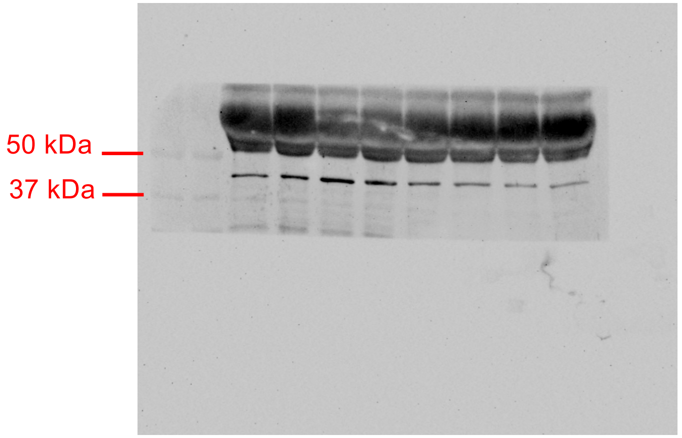

anti CD47

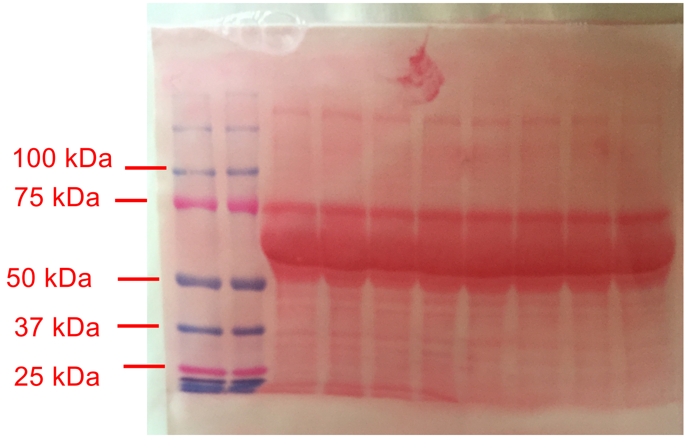

Ponceau S

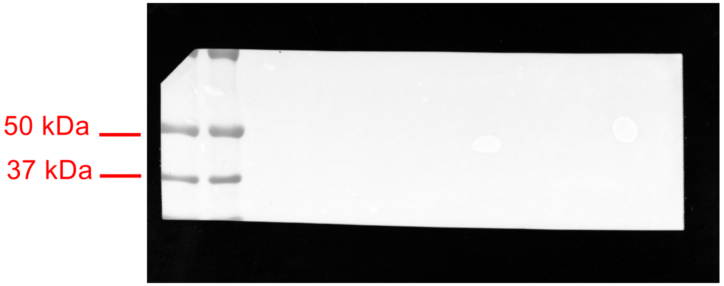

Colorimetric
(not shown in the manuscript)
