# Supplementary material for: CXCR4 engagement triggers CD47 internalization and antitumor immunization in a mouse model of mesothelioma
Source: EMBO Mol Med. 2021 May 6;13(6):e12344. doi: 10.15252/emmm.202012344 (PMC8185548; doi:10.15252/emmm.202012344)

Figure EV4 D

D Anti-HMGB1

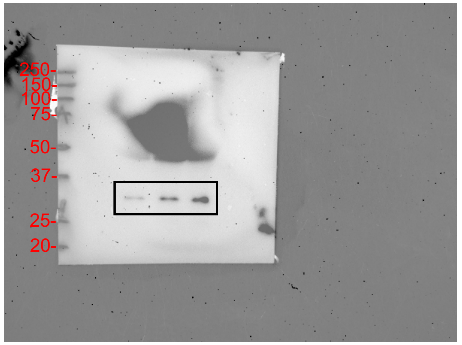

Duplicate not shown in the figure but used for statistical analysis

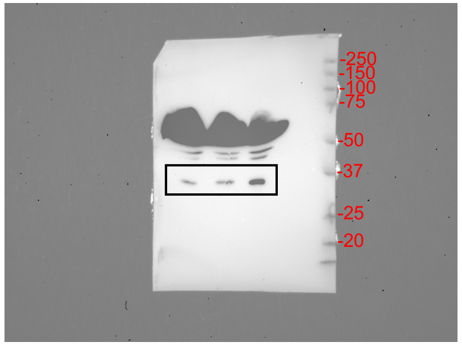

Anti-HMGB1

Ponceau not shown in the figure but it used for protein loading normalization

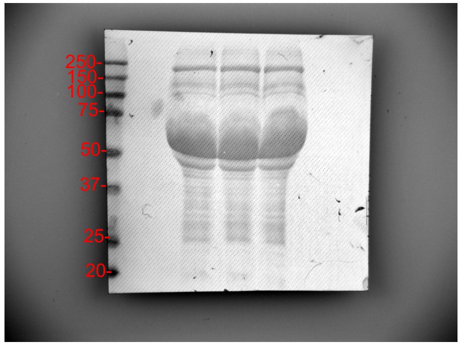

Ponceau (duplicate experiment) not shown in the figure but used for protein loading normalization

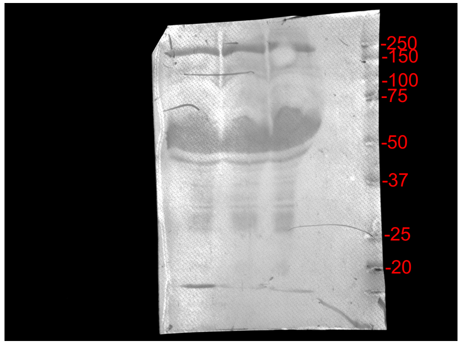

Figure EV4 E

p-eIF2α

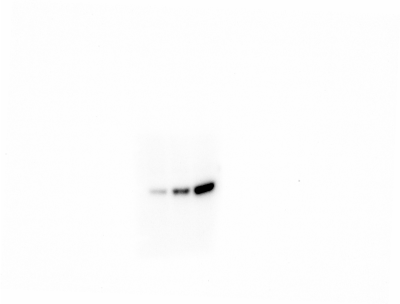

eIF2α

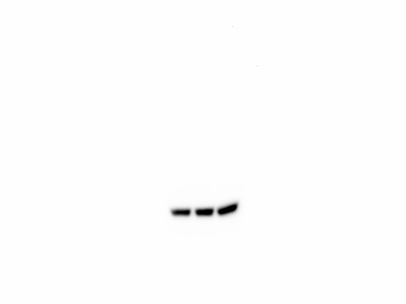

β-actin

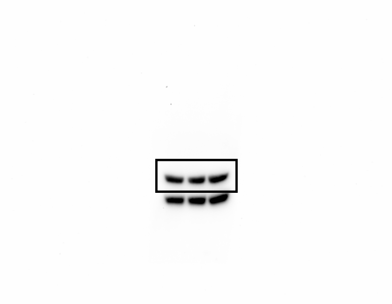

Exp1 (not shown in the figure but used for the statistic)

p-eIF2α

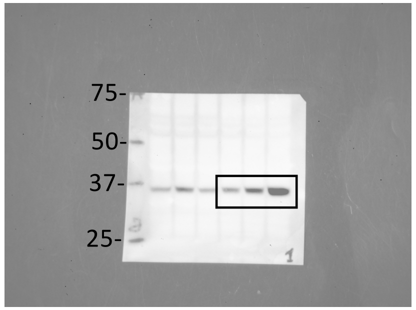

eIF2α

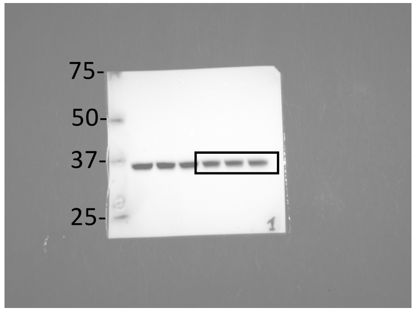

β-actin = IRE1

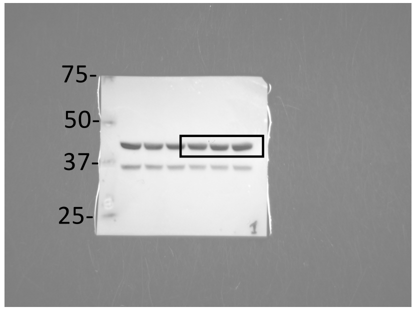

exp2

p-eIF2α

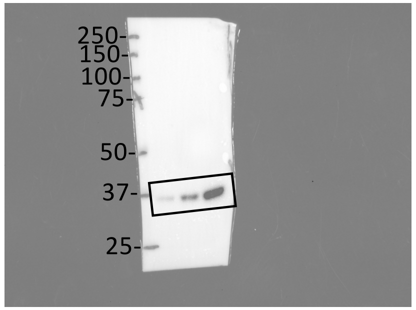

eIF2α

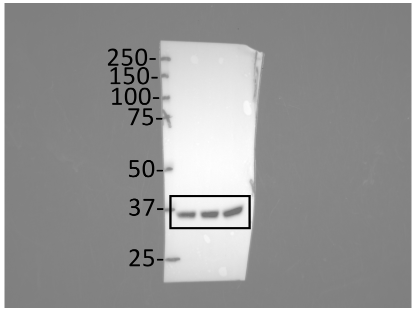

β-actin

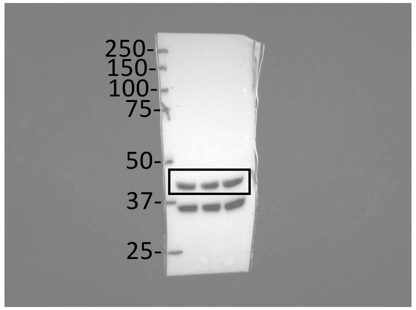

Figure EV4 E

exp1 **Exp2** (not shown in the figure but used for the statistic)

ATF6 (N)

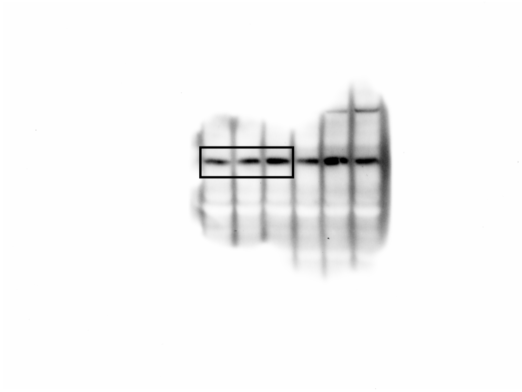

ATF6 (N)

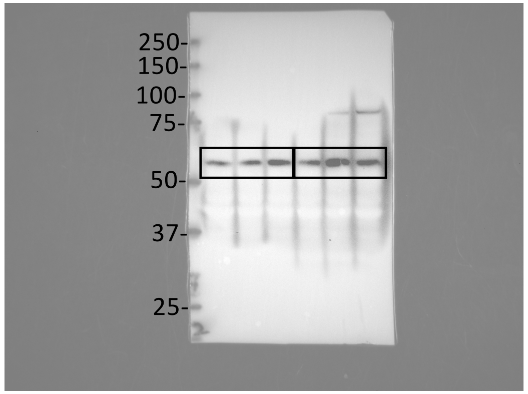

β-actin

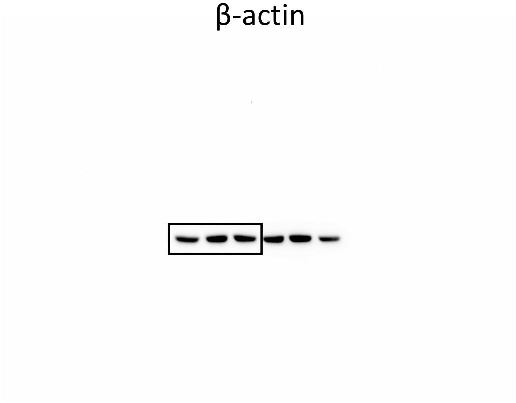

β-actin

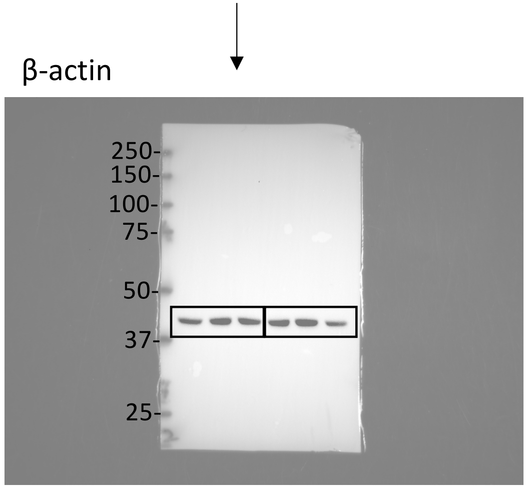

Figure EV4 E

Exp1 **Exp2** (not shown in the figure but used for the statistic)

p-IRE1 and IRE1

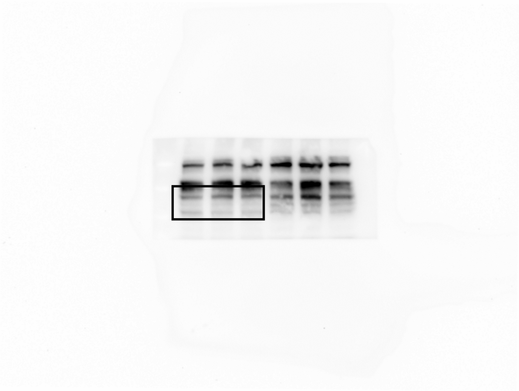

p-IRE1 and IRE1

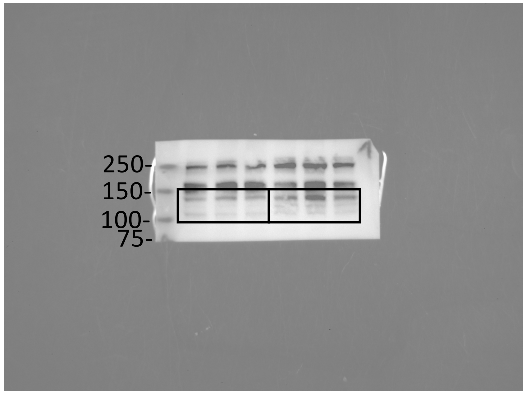

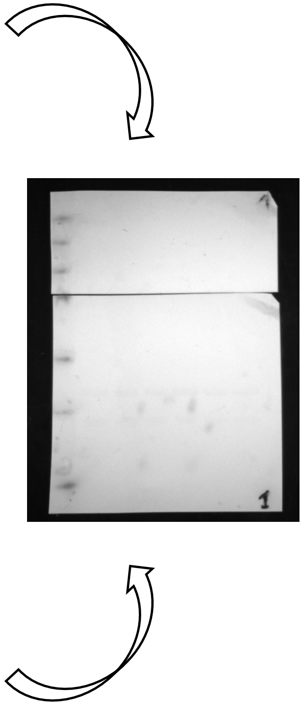

β-actin

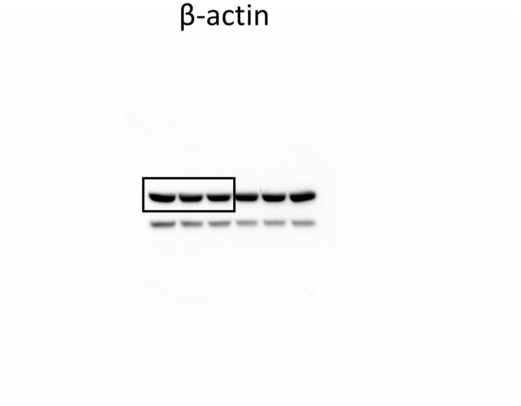

β-actin

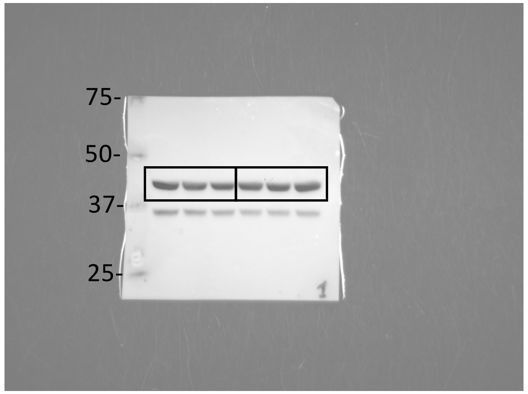

Supplement: Supplementary file 4 — Source Data for Expanded View and Appendix [file EMMM-13-e12344-s008.zip › EMM-2020-12344-V3-EV_Figures_SourceData/Source_of_data_EV4.zip!/Source of data EV4.pdf]
