# Supplementary material for: CXCR4 engagement triggers CD47 internalization and antitumor immunization in a mouse model of mesothelioma
Source: EMBO Mol Med. 2021 May 6;13(6):e12344. doi: 10.15252/emmm.202012344 (PMC8185548; doi:10.15252/emmm.202012344)
Supplement: Supplementary file 4 — Source Data for Expanded View and Appendix [file EMMM-13-e12344-s008.zip › EMM-2020-12344-V3-EV_Figures_SourceData/Source_of_data_EV5.zip!/Source of data EV5.pdf]

Figure EV5

B

Anti ATF6(N)   Anti β-actin

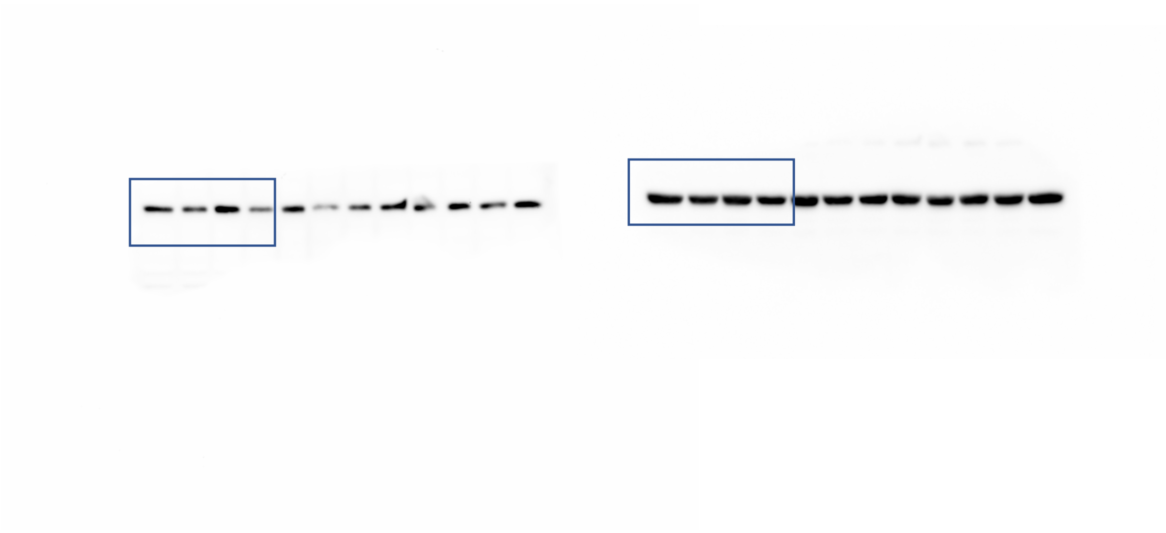

exp1 **exp2** **exp3**

ATF6 (N)

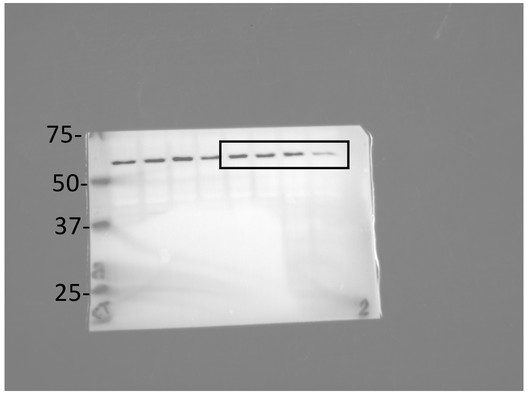

ATF6 (N)

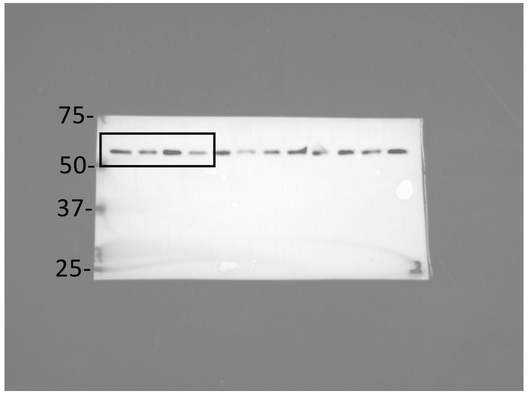

ATF6 (N)

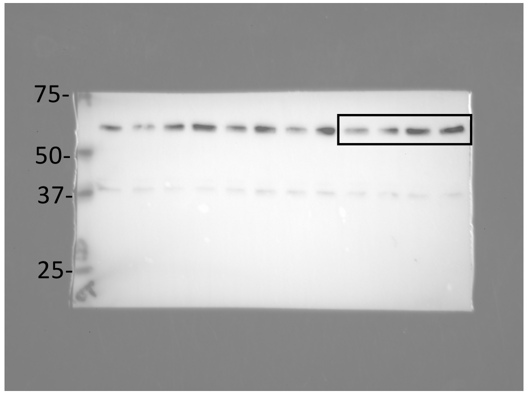

β-Actin

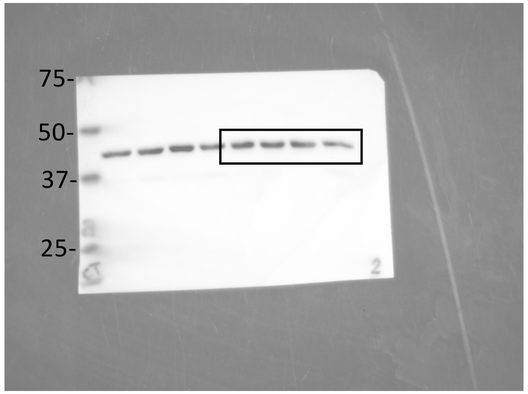

β-Actin

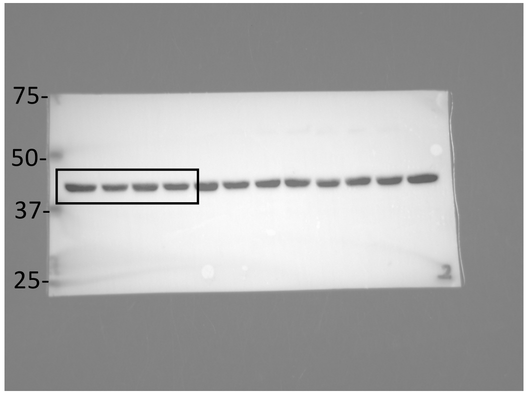

β-Actin

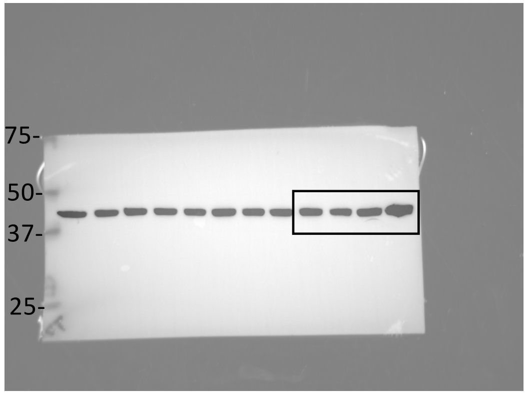

Exp 1 and 3 are not shown in the manuscript but they were used for the statistical analysis

Figure EV5

C

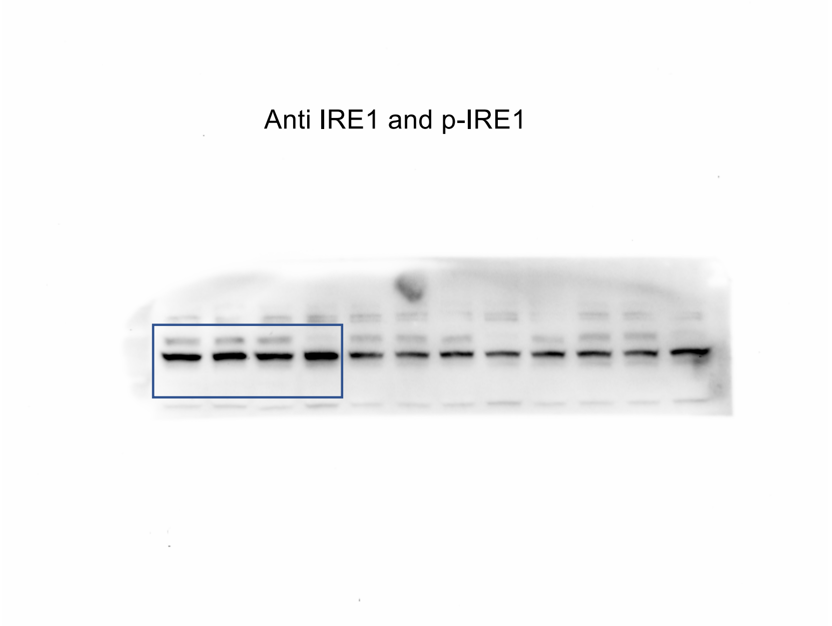

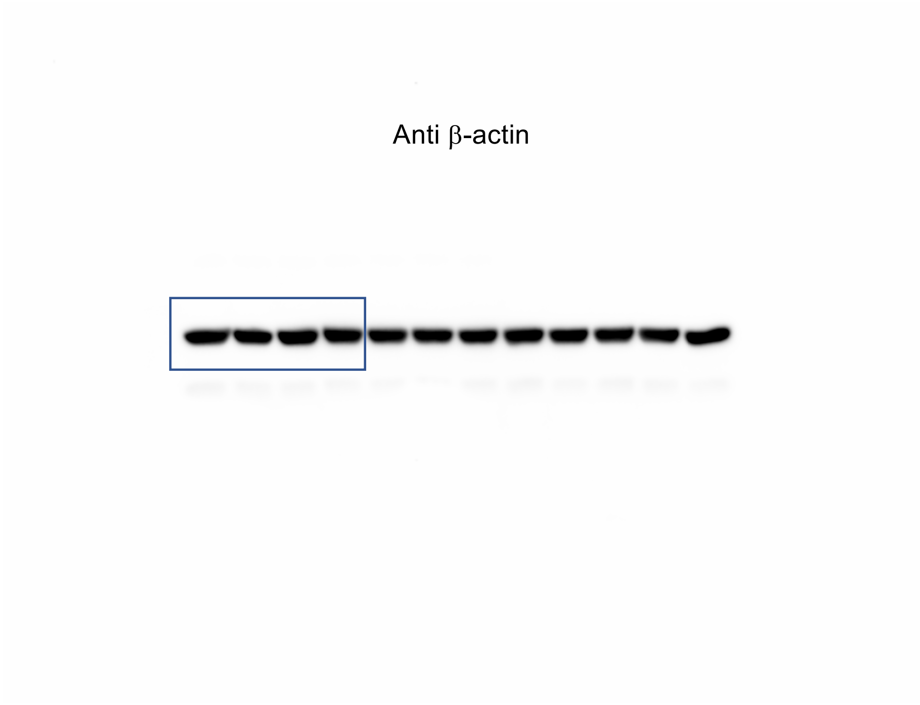

exp1 **exp2**

p-IRE1 and IRE1

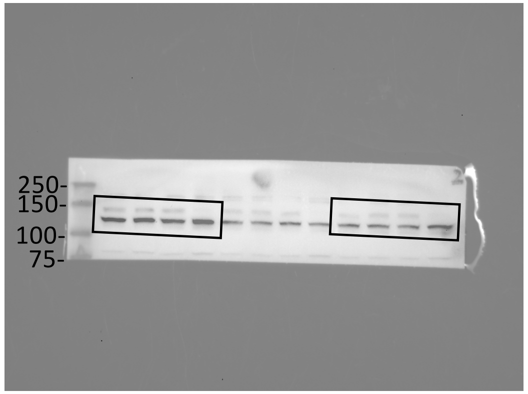

β-Actin

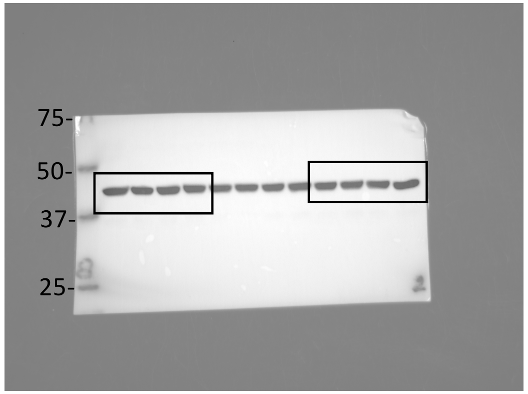

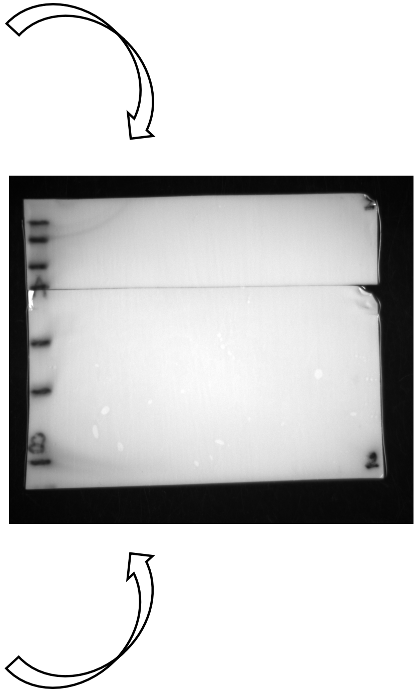

exp3

p-IRE1 and IRE1

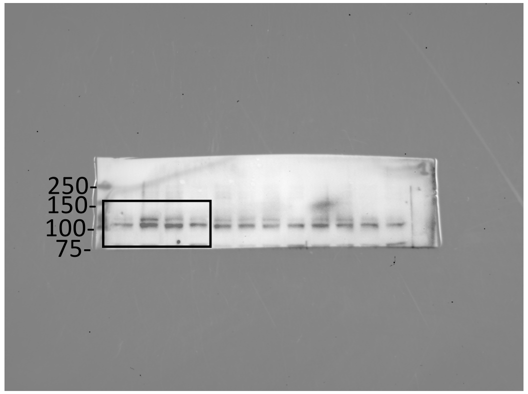

β-Actin

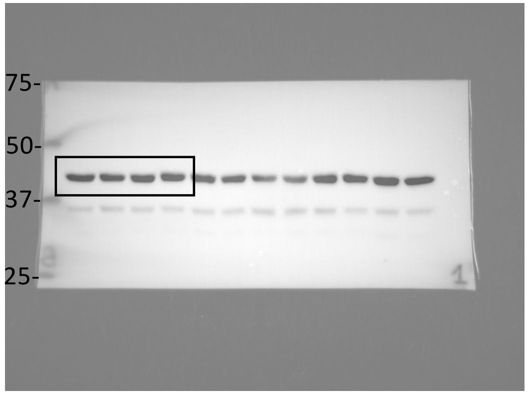

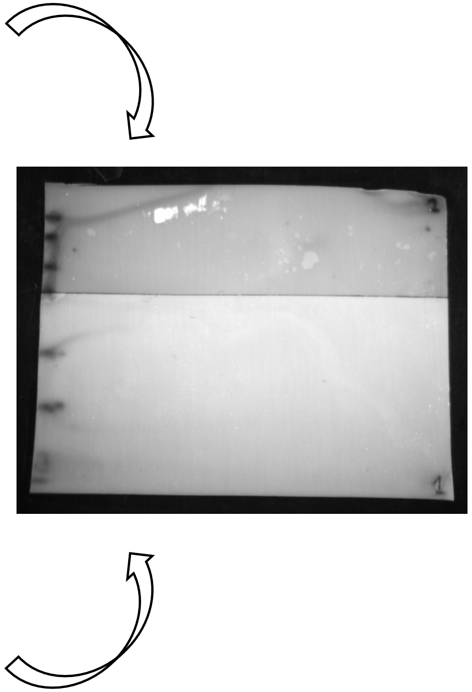

Exp 2 and 3 are not shown in the manuscript but they were used for the statistical analysis
